# Screening test accuracy of portable devices that can be used to perform colposcopy for detecting CIN2+ in low- and middle-income countries: a systematic review and meta-analysis

**DOI:** 10.1186/s12905-020-01121-3

**Published:** 2020-11-16

**Authors:** Katayoun Taghavi, Eliane Rohner, Partha Basu, Nicola Low, Anne Rutjes, Julia Bohlius

**Affiliations:** 1 Institute of Social and Preventive Medicine, University of Bern, Mittelstrasse 43, 3012 Bern, Switzerland; 2 The Graduate School for Cellular and Biomedical Sciences, University of Bern, Bern, Switzerland; 3 Screening Group, Early Detection and Prevention Section, International Agency for Research on Cancer, Lyon, France

**Keywords:** Sensitivity, Specificity, Low- and middle-income countries, Colposcopy, Cervical cancer screening

## Abstract

**Background:**

Portable devices that can be used to perform colposcopy may improve cervical cancer screening in low- and middle-income countries (LMIC) where access to colposcopy is limited. The objective of this study was to systematically review the diagnostic test accuracy (DTA) of these devices for the detection of cervical intraepithelial neoplasia grade 2 or higher (CIN2+).

**Methods:**

In accordance with our protocol (Prospero CRD42018104286), we searched Embase, Medline and the Cochrane Controlled Register of Trials up to 9/2019. We included DTA studies, which investigated portable devices with moderate-to-high optical magnification (≥ 6×) for colposcopy, as described in the manual for Colposcopy and Treatment by the International Agency for Research on Cancer, with a histopathological reference standard. We used the QUADAS-2 tool to assess study quality. We examined results for sensitivity and specificity in paired forest plots, stratified by stages in the clinical pathway. We pooled estimates of test accuracy for the index test, used as an add-on to other tests, using a bivariate random-effect model.

**Results:**

We screened 1737 references and assessed 239 full-text articles for eligibility. Five single-gate DTA studies, including 2693 women, met the inclusion criteria. Studies evaluated two devices (Gynocular™ and Pocket) at different stages of the screening pathway. In three studies, which used the index test in an add-on capacity in 1273 women, we found a pooled sensitivity of 0.79 (95% CI 0.55–0.92) and specificity of 0.83 (95% CI 0.59–0.94). The main sources of bias were partial verification, incorporation and classification bias.

**Conclusion:**

Few studies have evaluated portable devices able to perform colposcopy, so their accuracy for the detection of CIN2+ remains uncertain. Future studies should include patient-relevant and long-term outcomes, including missed cases, overtreatment, residual and recurrent disease. To meet the challenge of eliminating cervical cancer in LMIC, methods for visual assessment of the cervix need urgent redress.

## Background

The World Health Organization has called for coordinated global action to eliminate cervical cancer [1]. To achieve this goal, effective cervical screening in low- and middle-income-countries (LMIC), where 90% of women with cervical cancer live [2], is paramount. Screening strategies, which differ markedly between high- and low-income countries, may contribute to this inequity. Systemic challenges of high costs, limited healthcare infrastructure for laboratory dependent screening tests, transportation and electricity constraints, and limited specialists compromise the effectiveness of screening programs in LMIC. Currently, cervical cancer screening in many LMIC is based on the cheapest method, visual assessment with acetic acid (VIA), with screening and treatment on the same day. Efforts to improve cervical cancer screening strategies in LMIC must consider their feasibility in relation to systemic factors.

Despite huge advances in cervical cancer screening methods, including new molecular methods like human papillomavirus (HPV) testing [3], visual assessment of the cervix remains essential for screening for pre-cancerous lesions. In high-income countries, colposcopy methods remain fundamentally important in the screening pathway [4]. Colposcopy is an advanced method of visual inspection that allows detailed assessment of the cervix [5]. A full colposcopy examination, as described in the manual for Colposcopy and Treatment by the International Agency for Research on Cancer (IARC), includes assessment of the cervix with low- and high magnification of at least 6–15×, assessment with acetic acid, Lugol’s iodine, assessment with white and/or green light [5]. Colposcopy assessment in high-income settings is used both to direct biopsies and to make treatment decisions, which rely on accurate assessment of the site and size of a lesion. High-income countries, which have had the greatest success in reducing the burden of cervical cancer, employ a multi-step pathway of screening, treatment and follow-up [6, 7]. Colposcopy is usually performed after a positive screening test(s), as an ‘add-on’ test. The population receiving colposcopy therefore has a higher disease prevalence than the population receiving the first test (‘first-line’ test) in the screening pathway. Furthermore, women wait for the results of their biopsies and only women with histopathologically confirmed disease are treated.

Extensive screening and treatment pathways that require multiple clinic visits are not feasible in most LMIC. Stationary colposcopy, HPV testing, Papanicolaou (PAP) smears and histopathological confirmation are generally not used. Currently, in most LMIC a naked eye examination (VIA) is used for screening and treatment. In Africa, healthcare professionals with varying expertise often perform screening and studies report a wide range of sensitivity for VIA from 25.0 (95% CI 7.1–59.1) [8] to 94.4% (95% CI 84.6–98.8) [9]. The scale up of screening programs in LMIC could benefit from improved methods of visual assessment, particularly as an add-on to high-risk types of HPV (HR-HPV) testing, which can detect earlier stages of disease that are more difficult to detect with the naked eye. Portable devices that perform the functions of stationary colposcopes could improve visual assessment of the cervix in settings with fluctuating electricity supplies and inconsistent maintenance, particularly for mobile clinic services. The IARC manual for Colposcopy and Treatment of Cervical Intraepithelial Neoplasia defines 6× optical magnification as the minimum required for most of the work of colposcopy [5]. Optical magnification is the magnification that is achieved by the lens used. Portable devices may use digital zoom to enlarge an image captured by optical magnification. Such digital enlargement reduces the image resolution and clarity*.* Portable devices with only low optical magnification (eg < 6×) have not been shown to improve the detection of cervical neoplasia, beyond what is achievable by VIA alone [10]. The objective of this study was to evaluate portable devices that could be used to perform colposcopy for the detection of histologically confirmed cervical intraepithelial neoplasia, grade 2 or higher (CIN2+).

## Methods

We performed a systematic review of diagnostic test accuracy (DTA) studies. The study protocol is registered (Prospero CRD42018104286) (Additional file 1) and aligned with the Preferred Reporting Items for Systematic Reviews and Meta-Analyses for Diagnostic Test Accuracy Studies (PRISMA-DTA) [11]. We report our findings in accordance with these recommendations and include the checklist items.

### Eligibility criteria

We included studies assessing portable devices that can be used to perform colposcopy (index test) with at least 6× optical magnification. The colposcopic procedure had to meet standard colposcopy guidelines, as described above [5]. We only included studies evaluating devices that were mobile, not reliant on electricity, and could be used and maintained in LMIC. We excluded devices that achieved the required magnification by digital zoom, rather than optical magnification, or that assessed the tissue of the transformation zone and are used as alternatives to histology (also referred to as “visual biopsy” devices). As the reference standard, we required punch or excision biopsies for determining the presence of CIN2+.

Eligible study designs were: single-gate studies, with single inclusion criteria for participants, such as cross-sectional studies and cohort studies [12, 13]; multiple-gate studies, with two or more sets of inclusion criteria, such as case–control studies; randomised controlled trials and cohort studies that compared the persistence or recurrence of disease after a test-treat scheme.

### Search strategy

We searched Ovid Embase, Ovid Medline, Cochrane Central Register of Controlled Trials, ClinicalTrials.gov, and the Food and Drug Administration (FDA) website for eligible studies and conference abstracts. We performed the first search on the 5th March 2018, and an update on September 5th, 2019. Our search terms included “cervical cancer, pre-cancer”, “mass screening, early detection of cancer”, “colposcopes, alternate colposcopes” [14], and “mobile, point of care systems, telemedicine, mhealth”. We present the full Ovid Medline search strategy in Additional file 2. We identified additional studies through backward and forward citation searching of relevant articles. We did not apply any language restrictions. Two reviewers (KT and ER) independently screened titles and abstracts for relevance. Disagreements were resolved by consensus or through discussion with a third reviewer (JB). We applied the same method to assess eligibility of full-text manuscripts.

### Data extraction

One reviewer (KT) extracted the data into a piloted and standardised form. Another reviewer (ER) checked the data. Disagreements were resolved by consulting a third reviewer (JB) and reaching consensus. We extracted data on: study characteristics (setting, country, study year, publication year, study design); criteria for inclusion and exclusion; participant characteristics (age, education, smoking status, menopausal status, parity, HIV status); the index test (model, experience of the practitioner using the device, number of practitioners using the device, number of eligible women getting index test, number who received index test, explanations for discrepancies between those eligible and receiving the index test); the reference standard (reference standard, those eligible to receive reference standard, number who received reference standard, explanation for discrepancies between in those eligible and those receiving the reference standard); and the reported estimates of DTA with confidence intervals. Where possible, we extracted the absolute numbers of true positives, false negatives, false positives, and true negatives. If these numbers were not reported, we derived them from reported estimates of test accuracy, total number of included women, and prevalence. We assessed performance characteristics of eligible devices at different levels of severity using the Swede score, where available. The Swede score uses five parameters (vessels, margins or surface, acetic acid uptake, iodine staining and lesion size) to standardise the visual assessment of cervical lesions [15]. Each parameter is scored between zero and two, based on severity of the findings, and summed to a total score between zero (best) and ten (worst).

### Quality assessment

We used the Quality Assessment of Diagnostic Accuracy Studies (QUADAS-2) checklist to assess the quality of the included studies [16]. We defined the risk of partial verification bias as low if 10% or fewer women did not receive the reference standard test.

### Statistical analysis

We displayed sensitivity and specificity estimates in paired forest plots, for each test done, with corresponding 95% confidence intervals. Where the Swede score was used, we displayed estimates stratified by each Swede score threshold. We described the Swede score optimising sensitivity and specificity in each study. For the pooled sensitivity and specificity, we used the Swede score threshold of five, which is recommended as the cut-off optimising both sensitivity and specificity [17]. We pooled estimates of test accuracy when used as an add-on test (i.e. after a positive screening test) using a bivariate random-effect model [18]. We present this graphically with a hierarchic summary receiver-operating characteristic (HSROC) and describe the summary point, area under the receiver operating curve (AUC), 95% confidence and prediction contours. We used STATA 14 and RevMan 5.0.18 for these analyses.

## Results

### Literature search overview

**Fig. 1:**
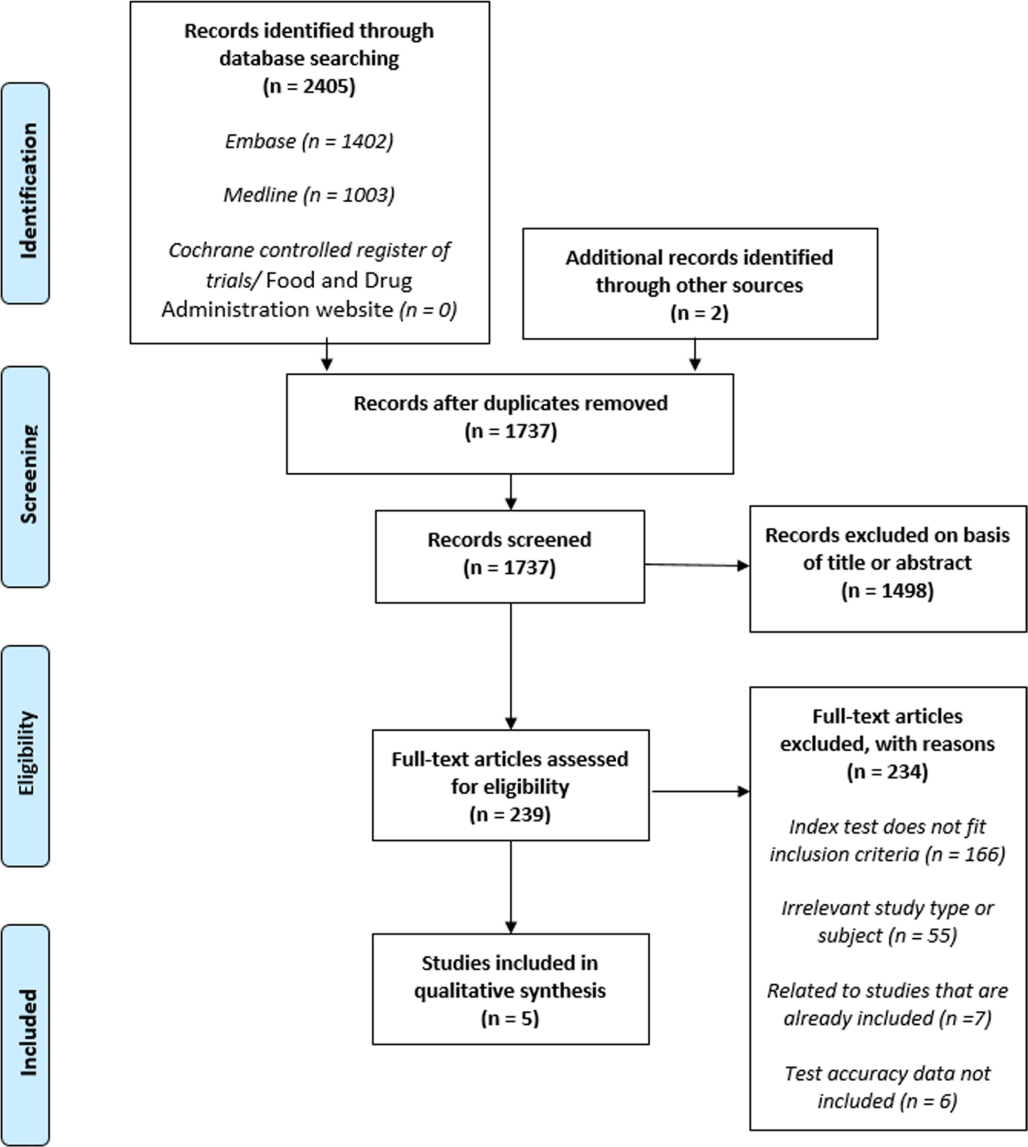
PRISMA flow diagram of articles evaluated for inclusion and exclusion. From: Moher D, Liberati A, Tetzlaff J, Altman DG, The PRISMA Group (2009). Preferred Reporting Items for Systematic Reviews and Meta-Analyses: The PRISMA Statement. PLoS Med 6(7): e1000097

Our literature search identified 1737 unique references. After screening titles and abstracts, we excluded 1498 citations and assessed the full-text of the remaining 239 articles. We excluded 234 studies (Fig. 1). Most excluded studies were ineligible because the index test did not fit our criteria (n = 166). We excluded 23 studies of stationary colposcopes, 30 studies of devices with less than 6× optical magnification (VIA and visual inspection with Lugol’s iodine, smartphones, EVA™, Aviscope™, cervicscan, and Magnivisualiser™), 21 studies where the full colposcopy procedure was not carried out (e.g. only acetic acid was used as with digital cervicography devices, smartphones, microscopes) and 92 studies of visual biopsy devices (e.g. artificial intelligence technologies, electrical impedance spectroscopy, confocal microscopy, Truscreen™, and sonoelastography). Six publications were ineligible because test accuracy data were missing [19–24]. Seven publications were based on study populations already included in our analysis [17, 25–29]. We have presented a complete list of excluded full-text assessments and the reasons for their exclusion in Additional file 3.

We included five single-gate diagnostic test accuracy studies [12]. Table 1 shows the characteristics of these studies, which include 2693 women. Four of the studies were conducted in LMIC (India [30], Bangladesh [31], Peru [32] and China [29]) and one was conducted in a high-income country (Sweden [33]). One study estimated DTA with two methods of screening [29] and another, for two different groups of providers (nurses/doctors) [31]. Four studies evaluated the Gynocular™ [30, 31, 33, 34] and one study evaluated the Pocket device. These devices have 4–12× and 3–30× optical magnification, respectively. All studies carried out the full colposcopy procedures outlined in the IARC manual for Colposcopy and Treatment of Cervical Intraepithelial Neoplasia [5]. Investigators from two studies obtained funding from the manufacturer for their contribution to the study. In all other studies where funding was obtained, the manuscript states that the funder did not play a role in planning and conducting research, or writing the manuscript.

**Table 1:** Characteristics of included studies: all obtain biopsy for identification of CIN2+

First author /publication year	Clinical setting	Index test	Procedure as described in manuscript	Age mean (SD)	Number of women receiving index test	Number of women receiving biopsy *(%)*	Number of women refusing biopsy	Biopsy indication	Person performing index test	Prior tests	Prevalence of CIN2+ n (%)	Funding
First-line test												
Newman 2019	Boashan, China	Gynocular™	Colposcopy *traditional *Use of green-filter not specified	44.3 **(6.7)	488	31 *(6.3)*	NR	Abnormal findings on colposcopy or abnormal cytology	Gynaecologists (1 of 2)	None	3/488* (0.6%)	Two devices were donated to the study
Mixed use: first-line test and add-on test												
Nessa 2014 Doctors	Dhaka, Bangladesh	Gynocular™	Colposcopy *IARC guidelines*	35.1 (8.1)	932	*228 (24.5)*	*28*	Women who had a Swede score of greater than 4	Gynaecologists or Colposcopy trained Physicians (1 of 6)	VIA (n = 528) OR no screening (n = 404)	39/932* (4.2%)	Two investigators were funded by the device manufacturer
Nessa 2014 Nurses	Dhaka, Bangladesh	Gynocular™	Colposcopy *IARC guidelines*	35.1 (8.1)	932	*228 (24.5)*	*28*	Women who had a Swede score of greater than 4	Colposcopy trained nurses (1 of 2)	VIA (n = 528) OR no screening (n = 404)	39/932* (4.2%)	Two investigators were funded by the device manufacturer
Add-on test only												
Banerjee 2018	West Bengal, India	Gynocular™	Colposcopy *IARC guidelines*	39.2 **(7.4)	1021	*1020 (99.9)*	1	All women who had the index test	Gynaecologist (uncertain how many gynaecologists were performing the index test)	HPV AND /OR VIA (180 had VIA only)	36/1021 (3.5%)	No funding
Kallner 2015	Stockholm, Sweden	Gynocular™	Colposcopy *IARC guidelines*	33.4 (9.9)	123	*113 (92.0)*	NR	Women who had a Swede score of greater than 0	Gynaecologist (1 of 6)	PAP smear AND HPV	44/123* (35.7%)	One investigator was funded by device manufacturer
Mueller 2018	Lima, Peru	Pocket	Colposcopy *Excluded green filter as not standard practice in Lima*	37.1 *** (20–67)	129	81 *(62.7)*	NR	Abnormal findings on colposcopy	Physicians (1 of 4)	HPV OR PAP smear	22/129* (17.1%)	Two National Institutes of Health grants

CIN2+, cervical intraepithelial neoplasia, grade two and above; Numbers in italics: calculated from data extracted; HPV, human papillomavirus; PAP smear, Papanicolaou smear test; VIA, visual inspection with acetic acid; IARC, International Agency for Research on Cancer; NR, not reported; SD, standard deviation

*Prevalence is based on assumption that all Women without biopsy were free from CIN2+

**SD approximated, based on data from age categories

***Age range, as reported in the pape

The studies evaluated test accuracy at different stages in the screening pathway. The Pocket device was evaluated as an add-on test to HPV or PAP-smear [24]. The Gynocular™ was evaluated as a first-line test [29] and as an add-on test to HPV, PAP-smear or VIA [30, 33]. In one study, the Gynocular™ device was used indiscriminately as a first-line test among 404 women (43%), and as an add-on test after VIA positivity among 528 women (57%) [31]. Estimates of test accuracy were not available separately for the two subgroups, so the results could not be summarised with the other study results. In studies assessing devices in an add-on capacity, disease prevalence ranged between 3.5% [30] and 35.7% [33]. In these studies, the colposcopic procedure followed a positive PAP smear and/or HPV and/or VIA test. Prevalence of CIN2+ in studies assessing the device and colposcopic procedure as a first-line test was 0.6% [29], and 4.2% [31] when used in either situation at two points in the screening pathway.

### Test accuracy for the detection of CIN2+

**Fig. 2:**
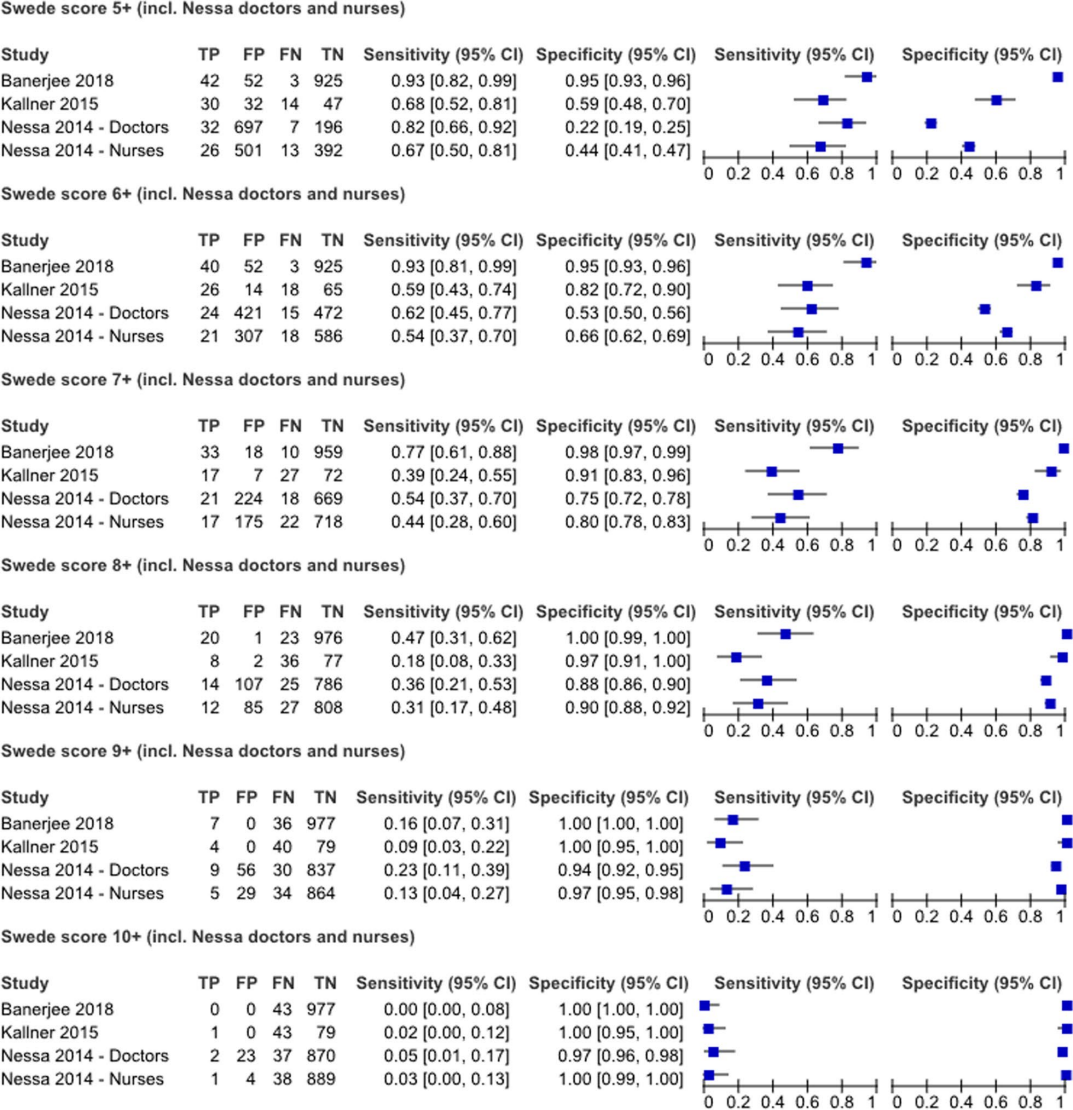
Paired forest plot for Swede scores five to ten. *TP* true positive, *FP* false positive, *FN* false negative, *TN* true negative, *CI* confidence intervals

Three of the four studies evaluating the Gynocular™ used the Swede scoring system to describe the colposcopy result [15]. We report sensitivity and specificity estimates for Swede score thresholds five and above (Fig. 2) and for all scores in Additional file 4. Across all studies, sensitivity decreased as Swede score threshold increased, and specificity increased. The Swede score that optimised sensitivity and specificity was calculated to be six in three studies in which doctors did the assessment [30, 31, 33], and seven in one study, where nurses did the assessment [31].

**Fig. 3:**
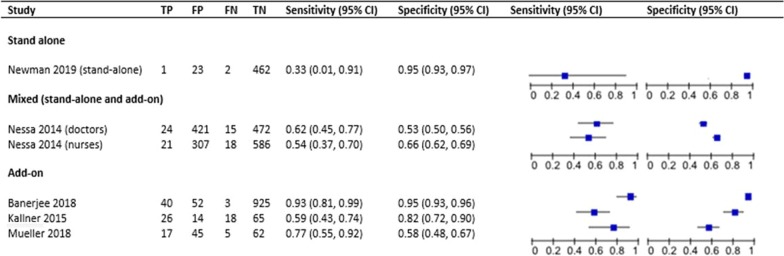
Paired forest plot of index test sensitivity and specificity stratified by clinical pathway. *TP* true positive, *FP* false positive, *FN* false negative, *TN* true negative, *CI* confidence intervals

**Fig. 4:**
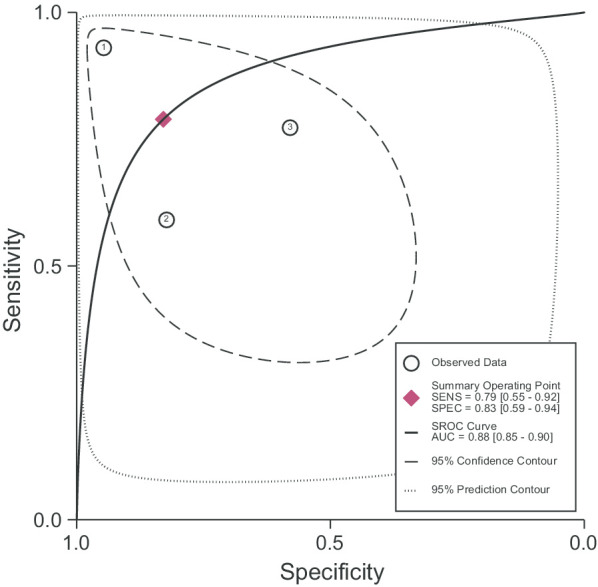
Bivariate model plot of add-on tests. 1, Banerjee 2018; 2, Kallner 2015; 3, Mueller 2018; SENS, sensitivity; SPEC, specificity; AUC, area under the receiver operating curve; SROC, summary receiver-operating characteristic

Figure 3 shows study estimates for sensitivity and specificity, stratified by stage in the clinical pathway. For each specific point, there were few studies. We pooled results from three studies, including 1273 women, which used the index test as an add-on to any previous test. We found a sensitivity of 0.79 (95% CI 0.55–0.92) and a specificity of 0.83 (95% CI 0.59–0.94), with an AUC of 0.88 (0.85–0.90) (Fig. 4). However, the prediction interval indicates a large degree of variation between studies and imprecision in the pooled estimate. One study reported sensitivity and specificity of the index test used as a first-line test, and found a sensitivity and specificity of 0.33 (95% CI 0.01–0.91) and 0.95 (95% CI 0.93–0.97), respectively [29]. We did not pool study estimates across different stages in the screening pathway.

### Quality assessment

Overall, the quality of the eligible studies was moderate. Assessment using the QUADAS-2 criteria identified three common areas that compromise studies in the domains of (1) patient selection, (2) index test, and (3) the reference standard Additional file 5.

In all five included studies, the sampling strategies were not detailed. It was unclear how the sample was derived, for example, whether a consecutive, random or convenience selection was used. Information about the target population was also missing, and no study reflected on whether the sample population was comparable to the target population. Data on excluded women were generally not available. In all studies, it was unclear whether selection bias influenced results.

Overall, the conduct of the index test was reasonable. However, in two studies (Nessa et al. [31] and Kallner et al. [33]), for 50% of women, the same assessor performed stationary colposcopy, followed immediately after by the index test. This sequence of events might have influenced the assessment of the index test. Several important issues regarding the reference standard were identified. Partial verification bias was identified infour out of five studies but considered to to have a high risk of bias in three. We considered two studies, Banerjee et al. and Kallner et al. [30, 33], to have a low risk of bias in the reference standard domain. In these studies, more than 90% of women who had received the index test also received the reference standard. In contrast, in Mueller et al. 63% of women received biopsy [32], in Nessa et al. 25% of women received biopsy [31], and in Newman et al., only 6% of women received a biopsy [29]*.* Conduct of the reference standard was problematic in two studies due to incorporation bias, where investigators use the index test to determine the need for reference standard and final diagnosis [31, 33]. These two studies used the Gynocular™ to assess Swede score, and used thresholds of 1 + [33] and 5 + [31] to determine if a biopsy was necessary. In contrast, two studies used alternative methods to indicate the need for biopsy. In Mueller et al., a standard colposcopic examination to determine the need for biopsy and by different assessors to those performing the index testing. In Newman et al.[29], of the 488 women who received the index test, 24 women were biopsied following Gynocular™ examination, and a further seven were biopsied following a positive HPV test, cytology and stationary colposcopic examination. As such, women who were negative for the index test in this study had alternative tests, reducing the risks of misclassification. None of the studies included verification of histopathological diagnoses as a method for quality control and minimising misclassification.

## Discussion

There are few diagnostic test accuracy studies of portable devices that can be used to perform colposcopy, so the sensitivity and specificity of such devices remains uncertain. The five studies that we identified examined the Gynocular™ and Pocket devices at different stages in the screening pathway. When used as an add-on screening test, the pooled sensitivity was 0.79 (95% CI 0.54–0.92) and specificity was 0.83 (95% CI 0.59–0.94). One study that used the Gynocular™ as a first-line test found a sensitivity and specificity of 0.33 (95% CI 0.01–0.91) and 0.95 (95% CI 0.93–0.97), respectively. The main sources of bias identified were partial verification, incorporation, and classification bias. Information about the target population and the selection of women was poorly reported, making it difficult to determine whether selection bias influenced findings.

The strengths of this systematic review are that we followed a pre-specified protocol, searched multiple electronic databases, systematically assessed quality of studies, and evaluated the DTA of the index test at different points of the screening pathway. We showed test accuracies for all Swede scores on paired forest plots. This allowed visualisation of the Swede score capacity to optimise either sensitivity or specificity, depending on the threshold used (Fig. 2 and Additional file 4).

The main limitation was that, owing to the small number of eligible studies, we were unable to do several of the planned analyses. There were too few studies to investigate heterogeneity, using regression methods, to assess test accuracy at different stages in the colposcopy screening pathway (first-line, mixed, or add-on), or the influence of preceding tests (eg. HPV test versus PAP smear). We found no longitudinal studies assessing test accuracy and its subsequent effects on patient-relevant outcomes such as overtreatment, residual and recurrent disease. Comparative systematic reviews of tests with relevant controls according to their intended place in the screening pathway will increase understanding of the use of a test in a particular population. This was beyond the scope of the present review.

Biases in the design of the included studies make interpretation of the findings uncertain. First, there was a high risk of partial verification bias in three of five studies [29, 31, 32], where less than 90% of index test recipients received the reference standard. Partial verification can result in overestimation of both sensitivity and specificity if women with more subtle disease are not identified. Second, we found evidene of incorporation bias, where the investigators used the index test to determine the need for the reference standard. This circularity may also artificially increase both the sensitivity and specificity of estimates. Third, classification bias, which describes how accurately true disease is identified, was noted. The reference standard of colposcopy-directed biopsy is the best available option for identification of true disease in the studies. More invasive reference standards, for example, excision of the transformation zone by cone biopsy or Loop Electrosurgical Excision Procedure (LEEP) would allow histological examination of the whole transformation zone, reducing the chance of misclassification, but carries unacceptable risks and potential long-term consequences for women of child-bearing age [35]. Newman et al. addressed potential misclassification of the reference standard by testing negative cases with alternative tests (HPV testing and stationary colposcopy) to minimise the risk of missing disease [29]. However, we were concerned about the small proportion of those receiving the index test who also received the reference standard. Other measures to minimise misclassification could be considered, such as obtaining more than one biopsy and obtaining biopsy in colposcopy-negative cases. These measures were not reported in any of the studies despite a large body of evidence to suggest that a single biopsy may miss true disease or underestimate disease prevalence [36–39]. Fourth, no studies reported on quality control or verification of histology results.

Taking into account the limitations of the studies in this systematic review, our findings on the accuracy of portable colposcopes used in an add-on capacity are consistent with current literature in most high-income settings [4, 14, 40]. We found a sensitivity of 0.79 (95% CI 0.55–0.92) and a specificity of 0.83 (95% CI 0.59–0.94), (AUC 0.88 [95% CI 0.85–90]) for portable devices that can be used to perform colposcopy as an add-on test. Many LMIC aim to provide single-visit screening and treatment for women, once or twice in their lifetime. With such few opportunities to see women, testing should rule-out disease in order that women will not miss the opportunity to be treated for pre-cancerous lesions of the cervix [42]. Ideally, screening with a highly sensitive first-line test should increase the prevalence of disease in the screened population before the next test is applied. As long as prevalence is low, the predictive value of a positive test also remains low [43]. In one study, where the Gynocular device was used as a first-line test, sensitivity was 0.33 (95% CI 0.01–0.91) [29]. At this level of sensitivity, based on the point estimate, portable colposcopes, as for stationary colposcopy, would not be useful as a first-line test. Furthermore, colposcopy is a specialized procedure and would be very resource intensive at this point of the screening pathway [40, 41]. We also found that the Swede score could be either highly sensitive or specific depending upon the threshold used. This supports the literature showing that scoring systems such as the Swede score can be used flexibly, to favour sensitivity or specificity, depending on the population and point in the screening pathway in which it is used [32].

We identified several alternatives and adjuncts to VIA, colposcopy and biopsy, though their technical specifications did not meet our inclusion criteria. We highlight some promising technologies for settings where skilled healthcare workers and laboratory facilities are scarce. Early studies on automated algorithms to evaluate cervigrams have found that CIN2+ can be identified with greater accuracy (AUC 0.91 [95% CI 0.89–0.93]) than original cervigram interpretation (AUC 0.69 [95% CI 0.63–0.74]) [44]. There are also emerging microscopy and spectroscopy devices (visual biopsy devices) that are mobile and may have potential in low-resource settings [45–48]. If evolving technologies eventually replace stationary colposcopy, these require robust evaluation, at defined stages in the screening pathway, and among the population in which they will be used.

To meet the challenge of eliminating cervical cancer in LMIC, studies exploring feasible methods to improve on current visual assessment strategies are urgently required. Our systematic review identifies information gaps and methodological issues that should be considered in future studies of cervical screening methods. First, the purpose of the test, the stage of use in the screening pathway, consequences to patients, and the resources available in the setting should be clear. These factors are specially important in the evaluating cervical cancer screening strategies because the purpose and consequences to patients differ significantly between high- and LMIC. In high-income countries, treatment follows biopsy confirmation of disease, whereas in LMIC treatment occurs in the absence of a confirmatory test, using an estimated risk of disease only**.** Second, randomised controlled trials should be used more often as they allow direct comparison of different screening strategies. Trials should be designed to assess short- and long-term patient-relevant outcomes including persistence or recurrence of disease. Third, methods to minimise bias in test accuracy studies should be considered. Protocols that require biopsies from most or all women are likely to increase the chance of correctly identifying cervical disease [36–39]. If this is not possible, and a study is sufficiently large, a random sample of low-risk patients who would not usually receive a biopsy could be selected for biopsy to estimate the fraction of false negatives. Statistical models for analysis of missing data that include all participants should lead to more valid estimates than simply assuming test negative results to be true negative results [49]. Methods to reduce misclassification should also be considered. For example, using multiple biopsies, composite reference standards, or following up on participants with another non-invasive screening test will improve the validity of the reference test. We stress the importance of designing studies where the index test does not determine the need for the reference standard. Quality control or verification for the interpretation of histological specimens should also be considered in future studies. The emergence of improved cervical cancer screening methods has not eliminated the need for visual assessment. Detection of HR-HPV, using nucleic acid amplification tests, allows identification of disease at an earlier stage than pre-existing strategies such as cytological assessment. The sub-optimal specificity of HR-HPV requires some form of triaging, either to direct biopsies or to make treatment decisions. So optical magnification, as a point-of-care add-on test, may be even more important. With the current challenges of visual inspection in LMIC, more studies on portable devices able to perform colposcopy are required. Our literature review found few portable devices that can be used to perform colposcopy so we cannot make recommendations about specific devices in scale-up efforts. However, considering the central role of colposcopy in the management of precancerous lesions, and despite the widespread scale-up of HR-HPV testing, more research in this area will be useful.

## Conclusion

We did a systematic review to determine the test accuracy of portable devices, with at least 6× optical magnification, that can be used for colposcopy and the detection of cervical neoplasia in LMIC. We found few studies and their results are heterogeneous. Future comparative studies are required to evaluate whether these devices improve patient-relevant outcomes including missed cases, overtreatment, and residual or recurrent disease in LMIC. To meet the challenge of eliminating cervical cancer in LMIC, methods for visual assessment of the cervix need to be improved urgently.

## Supplementary information

**Additional file 1**. “Protocol”. The protocol for the systematic review and meta-analysis.

**Additional file 2**. “Medline Ovid search strategy”. Description of the Medline Ovid search strategy.

**Additional file 3**. “Full text assessments_explanation for exclusions”. Table showing excluded full texts and explanations for their exclusion.

**Additional file 4**. “Paired forest plot for all Swede score studies”. Sensitivity and specificity estimates for all Swede score thresholds.

**Additional file 5**. “Quality of the eligible studies”. Quality of eligible studies is scored using the QUADAS-2 criteria.

## Data Availability

The datasets used and analysed during the present study are available from the corresponding author on reasonable request.

## Abbreviations

AUC: Area under the receiver operating curve
CI: Confidence interval
CIN2: Cervical intraepithelial neoplasia, grade two and above
DTA: Diagnostic test accuracy
FDA: Food and Drug Administration
HPV: Human papillomavirus
HSROC: Hierarchic summary receiver-operating characteristic
IARC: International Agency for Research on Cancer
LEEP: Loop Electrosurgical Excision Procedure
LMIC: Low- and middle-income countries
PAP: Papanicolaou
PRISMA-DTA: Preferred Reporting Items for Systematic Reviews and Meta-Analyses for Diagnostic Test Accuracy Studies
QUADAS: Quality Assessment of Diagnostic Accuracy Studies
VIA: Visual assessment with acetic acid

## References

[1] World Health Organization. WHO Director-General calls for all countries to take action to help end the suffering caused by cervical cancer. Geneva: World Health Organization; 2013. https://www.who.int/reproductivehealth/call-to-action-elimination-cervical-cancer/en/.

[2] Fitzmaurice C, Allen C, Barber RM, Barregard L, Bhutta ZA, Brenner H (2017). Global, regional, and national cancer incidence, mortality, years of life lost, years lived with disability, and disability-adjusted life-years for 32 cancer groups, 1990 to 2015. JAMA Oncol.

[3] World Health Organization. Guidelines for screening and treatment of precancerous lesions for cervical cancer prevention. Geneva: World Health Organization; 2013. https://apps.who.int/iris/bitstream/10665/94830/1/9789241548694_eng.pdf. 24716265

[4] Schiffman M, Wentzensen N (2015). Issues in optimising and standardising the accuracy and utility of the colposcopic examination in the HPV era. Ecancermedicalscience.

[5] International agency for research on Cancer. Colposcopy and treatment of cervical intraepithelial neoplasia: a beginners’ manual. https://screening.iarc.fr/doc/Colp oscopymanual.pdf.

[6] Arbyn M, Raifu AO, Weiderpass E, Bray F, Anttila A (2009). Trends of cervical cancer mortality in the member states of the European Union. Eur J Cancer.

[7] Anttila A, Ronco G, Clifford G, Bray F, Hakama M, Arbyn M (2004). Cervical cancer screening programmes and policies in 18 European countries. Br J Cancer.

[8] Bigoni J, Gundar M, Tebeu P-M, Bongoe A, Schäfer S, Fokom-Domgue J (2015). Cervical cancer screening in sub-Saharan Africa: a randomized trial of VIA versus cytology for triage of HPV-positive women. Int J Cancer.

[9] De Vuyst H, Claeys P, Njiru S, Muchiri L, Steyaert S, De Sutter P (2005). Comparison of pap smear, visual inspection with acetic acid, human papillomavirus DNA-PCR testing and cervicography. Int J Gynecol Obstet.

[10] Sankaranarayanan R, Shastri SS, Basu P, Mahé C, Mandal R, Amin G (2004). The role of low-level magnification in visual inspection with acetic acid for the early detection of cervical neoplasia. Cancer Detect Prev.

[11] McInnes MDF, Moher D, Thombs BD, McGrath TA, Bossuyt PM, Clifford T (2018). Preferred reporting items for a systematic review and meta-analysis of diagnostic test accuracy studies the PRISMA-DTA statement. JAMA.

[12] Rutjes AWS, Reitsma JB, Vandenbroucke JP, Glas AS, Bossuyt PMM (2005). Case-control and two-gate designs in diagnostic accuracy studies. Clin Chem.

[13] Dehmoobad Sharifabadi A, Leeflang M, Treanor L, Kraaijpoel N, Salameh J-P, Alabousi M (2019). Comparative reviews of diagnostic test accuracy in imaging research: evaluation of current practices. Eur Radiol.

[14] Hermens M, Ebisch RM, Galaal K, Bekkers RL (2016). Alternative colposcopy techniques: a systematic review and meta-analysis. Obstet Gynecol.

[15] Bowring J, Strander B, Young M, Evans H, Walker P (2010). The swede score. J Low Genit Tract Dis.

[16] Whiting PF, Rutjes AWS, Westwood ME, Mallett S, Deeks JJ, Reitsma JB (2011). QUADAS-2: a revised tool for the quality assessment of diagnostic accuracy studies. Ann Intern Med.

[17] Basu P, Banerjee D, Mittal S, Mandal R, Ghosh I, Das P (2016). Evaluation of a compact, rechargeable, magnifying device to triage VIA and HPV positive women in a cervical cancer screening program in rural India. Cancer Causes Control.

[18] Rutter CM, Gatsonis CA (2001). A hierarchical regression approach to meta-analysis of diagnostic test accuracy evaluations. Stat Med.

[19] Asiedu MN, Guillermo S, Ramanujam N (2017). Low-cost, speculum-free, automated cervical cancer screening: Bringing expert colposcopy assessment to community health. Ann Glob Health.

[20] Goldstein L, Goldstein A, Kellogg-Spadt S, Marfori C, Goldstein A (2017). 002 digital cervicography for quality control of visualization with acetic acid (VIA) for cervical dysplasia screening. J Sex Med.

[21] Krishnan L, Bapat A, Sakhilkar R, Raje S, Gaikwad A, Busheri L (2017). Telemedicine-based community screening of cervical cancer. Indian J Public Heal Res Dev.

[22] Lam CT, Mueller J, Asma B, Asiedu M, Krieger MS, Chitalia R (2018). An integrated strategy for improving contrast, durability, and portability of a Pocket Colposcope for cervical cancer screening and diagnosis. PLoS ONE.

[23] Ngonzi J, Bajunirwe F, Wistrand C, Mayanja R, Altman D, Thorsell M (2013). Agreement of colposcope and gynocular in assessment of cervical lesions by swede score: a randomized, crossover pilot trial. J Low Genit Tract Dis.

[24] Mueller JL, Asma E, Lam CT, Krieger MS, Gallagher JE, Erkanli A (2017). International image concordance study to compare a point-of-care tampon colposcope with a standard-of-care colposcope. J Low Genit Tract Dis.

[25] Nessa A, Wistrand C, Begum SA, Thuresson M, Shemer I, Thorsell M (2014). Evaluation of the cervical swede score method and the gynocular by colposcopy trained VIA nurses: a cross-over randomised trial. BJOG Int J Obstet Gynaecol.

[26] Nessa A, Wistrand C, Begum SA, Thuresson M, Shemer I, Thorsell M (2014). Evaluation of stationary colposcope and the gynocular, by the swede score systematic colposcopic system in VIA positive women a crossover randomized trial. Int J Gynecol Cancer.

[27] Taghavi K, Banerjee D, Mandal R, Kallner HK, Thorsell M, Friis T (2018). Colposcopy telemedicine: live versus static swede score and accuracy in detecting CIN2+, a cross-sectional pilot study. BMC Womens Health.

[28] Mueller JL, Lam CT, Kellish M, Peters J, Asiedu M, Krieger MS (2017). Clinical evaluation of a portable pocket colposcope for cervical cancer screening in the United States, Perú, and Tanzania. IEEE Healthc Innov Point Care Technol.

[29] Newman H, Hu J, Li X, He J, Bradford L, Shan S (2019). Evaluation of portable colposcopy and human papillomavirus testing for screening of cervical cancer in rural China. Int J Gynecol Cancer.

[30] Banerjee D, Taghavi K, Mandal R, Rohner E, Mittal S, Maji T (2018). Gynocular^TM^ as a field colposcope: real-life experiences from a VIA and HPV DNA-based cervical cancer screening program in Rural India. J South Asian Fed Menopause Soc.

[31] Nessa A, Roy JS, Chowdhury MA, Khanam Q, Afroz R, Wistrand C (2014). Evaluation of the accuracy in detecting cervical lesions by nurses versus doctors using a stationary colposcope and Gynocular in a low-resource setting. BMJ Open.

[32] Mueller JL, Lam CT, Dahl D, Asiedu MN, Krieger MS, Bellido-Fuentes Y (2018). Portable Pocket colposcopy performs comparably to standard-of-care clinical colposcopy using acetic acid and Lugol’s iodine as contrast mediators: an investigational study in Peru. BJOG An Int J Obstet Gynaecol.

[33] Kallner HK, Persson M, Thuresson M, Altman D, Shemer I, Thorsell M (2015). Diagnostic colposcopic accuracy by the gynocular and a stationary colposcope. Int J Technol Assess Heal Care.

[34] Newman H, Jilin H, Zhu B, Bradford L, Gao G (2019). Evaluation of portable colposcopy and HPV testing for screening of cervical cancer in rural China. Gynecol Oncol.

[35] Jin G, Lanlan Z, Li C, Dan Z (2014). Pregnancy outcome following loop electrosurgical excision procedure (LEEP) a systematic review and meta-analysis. Arch Gynecol Obstet.

[36] Baasland I, Hagen B, Vogt C, Valla M, Romundstad PR (2016). Colposcopy and additive diagnostic value of biopsies from colposcopy-negative areas to detect cervical dysplasia. Acta Obstet Gynecol Scand.

[37] Gage JC, Hanson VW, Abbey K, Dippery S, Gardner S, Kubota J (2006). Number of cervical biopsies and sensitivity of colposcopy. Obstet Gynecol.

[38] Wentzensen N, Walker J, Smith K, Gold MA, Zuna R, Massad LS (2018). A prospective study of risk-based colposcopy demonstrates improved detection of cervical precancers. Am J Obstet Gynecol.

[39] Wentzensen N, Walker JL, Gold MA, Smith KM, Zuna RE, Mathews C (2015). Multiple biopsies and detection of cervical cancer precursors at colposcopy. J Clin Oncol.

[40] Denny L, Quinn M, Sankaranarayanan R (2006). Chapter 8: screening for cervical cancer in developing countries. Vaccine.

[41] Denny LA, Sankaranarayanan R, De Vuyst H, Kim JJ, Adefuye PO, Alemany L (2013). Recommendations for cervical cancer prevention in Sub-Saharan Africa. Vaccine.

[42] Pewsner D, Battaglia M, Minder C, Marx A, Bucher HC, Egger M (2004). Ruling a diagnosis in or out with “SpPIn” and “SnNOut”: a note of cautiona note of caution. BMJ.

[43] Leeflang MMG, Rutjes AWS, Reitsma JB, Hooft L, Bossuyt PMM (2013). Variation of a test’s sensitivity and specificity with disease prevalence. CMAJ.

[44] Hu L, Bell D, Antani S, Xue Z, Yu K, Horning MP (2019). An observational study of deep learning and automated evaluation of cervical images for cancer screening. Obstet Gynecol Surv.

[45] Grant BD, Quang T, Possati-Resende JC, Scapulatempo-Neto C, de Macedo MG, Mauad EC (2019). A mobile-phone based high-resolution microendoscope to image cervical precancer. PLoS ONE.

[46] Parra SG, Rodriguez AM, Cherry KD, Schwarz RA, Gowen RM, Guerra LB (2019). Low-cost, high-resolution imaging for detecting cervical precancer in medically-underserved areas of Texas. Gynecol Oncol.

[47] Singhakum N, Laiwejpithaya S, Chaopotong P (2018). Digital cervicography by simply portable device as an alternative test for cervical cancer screening in rural area of Thailand. Asian Pac J Cancer Prev.

[48] Hunt B, Fregnani JHTG, Schwarz RA, Pantano N, Tesoni S, Possati-Resende JC (2018). Diagnosing cervical neoplasia in rural brazil using a mobile van equipped with *in vivo* microscopy: a cluster-randomized community trial. Cancer Prev Res.

[49] Naaktgeboren CA, de Groot JAH, Rutjes AWS, Bossuyt PMM, Reitsma JB, Moons KGM (2016). Anticipating missing reference standard data when planning diagnostic accuracy studies. BMJ.

