# Supplementary material for: Screening test accuracy of portable devices that can be used to perform colposcopy for detecting CIN2+ in low- and middle-income countries: a systematic review and meta-analysis
Source: BMC Womens Health. 2020 Nov 16;20:253. doi: 10.1186/s12905-020-01121-3 (PMC7670616; doi:10.1186/s12905-020-01121-3)
Supplement: Supplementary file 1 — Additional file 1. “Protocol”. The protocol for the systematic review and meta-analysis. [file 12905_2020_1121_MOESM1_ESM.docx]

Protocol: **Title: Diagnostic accuracy of Portable Colposcopic devices for detecting pre-cancerous cervical lesions: a systematic review protocol**

**Background:**

The burden of cervical cancer has significantly declined in high-income countries, where effective screening has reduced the incidence of cervical cancer by up to 80%[1]. In high-income settings, cervical cancer is now considered a rare disease. In contrast, it remains a public health priority in most low or middle income countries (LMIC), and remains the most common cause of cancer-related death for women[2].

Objective or automated screening tests, such as HPV testing are useful in contexts where both the number of specialists and access to them is limited. However, these screening strategies, in isolation are unlikely to solve the cervical cancer problem in LMIC. This is because it is essential that women with high-risk pre-cancerous lesions receive treatment that entirely removes their lesions. Otherwise screening programs will fail to prevent the progression of precancerous lesions to cancer. This hinges on accuracy of visual assessment prior administration of treatment. Therefore, this subjective assessment remains fundamental to cervical cancer screening and treatment.

The current evidence on efficacy of visual inspection after application of acetic acid (VIA), the most simple form of visual assessment, is poor. The sensitivity of VIA in Africa is reported to range from 25.0 (95% CI 7.1–59.1)[6] to 94.4% (95% CI 90.6 – 79.0)[7]. The main issue with VIA is that there is little that can be learned through the a VIA screening system as it is mostly set up today. It is inherently limited when not coupled with any other testing, such as histology, cytology or HPV and often lacks quality control mechanisms. To ensure a strong connection between screening and treatment, women are also assessed immediately for treatment with what is termed the “single visit approach”. Treatment decisions are usually made without histopathological confirmation of disease, without supervision, and often by nurses or midwives with varying expertise in the area. Studies have shown that women who receive inadequate treatment of precancerous disease are most likely to develop invasive cancer. In a recent 30 year retrospective cohort study including 1125 women with CIN3+ lesions who would have been eligible for minor local treatments, suboptimal treatment was associated with a cumulative incidence of invasive cancer of 40% (95% CI 20- 60%)[3]. There is also a body of evidence showing residual disease after treatment is strongly associated with future development of cervical smear abnormalities and CIN3+ lesions[4,5].

Colposcopy represents the gold standard for visual assessment of the cervix and has remained the cornerstone of cervical cancer prevention in high-income countries. This allows visualization of the cervix with magnification of 6x to 15x[6]. The examination includes assessment of the transformation zone with a green filter to identify abnormal vasculature, as well as detailed assessments using acetic acid and lugol solution to identify other well known stigmata of precancerous or earlier cancerous lesions. Recently, studies have examined the potential of a several portable colposcopic devices. These studies have not been evaluated together and given that the outcomes of interest, CIN2+ and cervical cancer are rare in the general population, it is useful to combine the data together.

**The objective is as follows**

1. To assess the diagnostic test accuracy of portable colposcopes, to detect underlying CIN2+ among adult women.

**Index test:**

Mobile colposcopy: In order to meet the criteria for a colposcope, the device must allow visualization of the cervix with 4-15X optical magnification. The device must be mobile, compact or hand held, and it must not dependent on an electricity supply.

**Methods**

**Search strategy**: This protocol was written in accordance with the Preferred Reporting Items for Systematic Reviews and Meta-Analyses (PRISMA) criteria. Embase, Medline, Cochrane Library, Clinical trials registry and FDA web site for mobile colposcopes will be searched upto final date of search, and additional studies will be located through backwards and forwards citation chaining.

The search terms used included “Cervical cancer / precancer” “Mass screening / early detection of cancer” “colposcopes/alternate colposcopes” “mobile / point of care systems / telemedicine / mhealth”. The search strategy for Medline is illustrated in Appendix 1. The literature search will identify studies without language restriction.

Two reviewers (KT and ER) will verify the inclusion and exclusion of studies independently and discuss if any discordances. If no consensus can be reached, a third review author (JB) will be consulted.

**Study eligibility:** The following criteria had to be fulfilled in order for articles to be included in the meta-analysis:

1. Study design: RCTs, cohort, cross sectional studies
2. Population: whenever mobile colposcopy could be used for cervical cancer screening purposes
3. Index test:
   - 1. Sufficient optical magnification (6-14X)
     2. Monocular and binocular acceptable
     3. Diagnostic procedure: visualization of cervix with high magnification, white light (+- green light) assessment, acetic acid, and lugols solution
     4. Device should be portable for use in the field settings
4. Reference standard: punch biopsy or excision biopsy (LOOP or LEEP) of the cervix. This is required in all participants, or all those considered to have a positive test should have the reference standard.
5. Target condition: histologically proven CIN2+
6. Pre-test: not restricted

**Data Extraction:** One author will extract the data (KT) and another person will fact-check it (ER). They will subsequently discuss in the case of differences. Where possible, the absolute numbers of true-positives, false-negatives, true-positives, true-negatives will be extracted directly from the article. Where accuracy parameters are reported but not the absolute numbers of true and false, positives and negatives, it will be derived or computed from the reported data. If this data still cannot be obtained, we will contact authors directly. If there is data for multiple thresholds of the index test, this will all be reported as above.

**Quality Assessment:** Methodological quality will be assessed against the Quality Assessment of Diagnostic Accuracy Studies checklist [7]. For each study, a methodological quality table will be completed. Table 1 explains how the domains will be interpreted by reviewers in order to achieve a score of “no-concerns”. Overall assessment is concluded by the sum of assessments in each domain (i.e. if 2 out of 3 questions were unclear, overall assessment for that domain will be unclear).

**Statistical anaylsis:** Using sensitivities and specifities as the main outcomes of interest, results from each study will be displayed on “paired” forest plots. Results will also be presented graphically on summary ROC (SROC) curves. Data for all thresholds of the index test will be reviewed. If appropriate we will perform a random-effects meta-analysis to derive the combined overall estimates of test accuracies. We may also explore causes of heterogeneity using meta-regression. This includes additional characteristics considered to be potentially influential to the accuracy of screening tests.. The following sources of between-study heterogeneity will be investigated

1. Study design and quality issues (QUADAS checklist)
2. Characteristics of the study population
3. Procedures of reference standard verification
4. Qualification of test assessors
5. Tests preceding the colposcopy examination (VIA, HR-HPV and PAP-smear)

**Table 1: Use of the Quality Assessment of Diagnostic Accuracy Studies checklist in this Review**

| **Quality domain** | **Definition** | **Comment** |
| --- | --- | --- |
| **1.Patient selection** | Consecutive or random sample of patients enrolled | No concerns if some information about the target population is also described, for example:   - Total number of target population   and   - Description of how sample population derived including the words “consecutive” or “random sample”. |
| **2.Index test** | Methods of assessing index test | No concerns, if the index test interpreted without knowledge of the reference |
|  |  | Also to distinguish in the data extraction:   - Were different thresholds used for interpretation of the index test - Was is pre-specified - Could the conduct or interpretation of the index test introduce bias (i.e. by knowledge of preceding tests) |
|  |  |  |
|  |  |  |
| **3.Reference standard**  3a. was partial verification avoided? | Histopathology obtained from colposcopy followed by a targeted biopsy will be the reference standard. If it is possible to further classify the biopsies as i) those that were targeted and ii) those taken when no lesion was seen, this will be distinguished in the data collection.  Histology results from excision biopsies will also be noted. | No concerns if all included participants received the same reference standard. However, depending on the colposcopy results, the policy of taking a biopsy may differ between studies. To elaborate further, if all participants did not receive the reference standard, the risk that this could have introduced bias will still be considered low if it affected 10% of the sample population or less. |
| 3b. was incorporation avoided | Was the reference standard independent of the index test (did the index test form part of the reference standard?). | No concerns if the reference standard was dependent on the index test. |
| 3c. blinding |  | No concerns if colposcopy and histological interpretation of biopsies were executed with or without knowledge of the index or comparator tests. |
| 3d. were uninterpretable results reported |  |  |
| **4. Flow and timing**  4a. acceptable delay between test | Normally the reference test should be done at the same time as colposcopy. | A delay between index test and reference standard will be recorded. |
| 4b. Withdrawals explained |  | No concerns if loss of patients, and missing or uninterpretable test results is explained. |

**References:**

1 Arbyn M, Raifu AO, Weiderpass E, *et al.* Trends of cervical cancer mortality in the member states of the European Union. Published Online First: 2009. doi:10.1016/j.ejca.2009.07.018

2 Fitzmaurice C, Allen C, Barber RM, *et al.* Global, Regional, and National Cancer Incidence, Mortality, Years of Life Lost, Years Lived With Disability, and Disability-Adjusted Life-years for 32 Cancer Groups, 1990 to 2015. *JAMA Oncol* 2017;**3**:524. doi:10.1001/jamaoncol.2016.5688

3 Paul C, Sharples KJ, Baranyai J, *et al.* Outcomes for women without conventional treatment for stage 1A (microinvasive) carcinoma of the cervix. *Aust New Zeal J Obstet Gynaecol* 2018;**58**:321–9. doi:10.1111/ajo.12753

4 Flannelly G, Bolger B, Fawzi H, *et al.* Follow up after LLETZ: could schedules be modified according to risk of recurrence? *BJOG* 2001;**108**:1025–30.http://www.ncbi.nlm.nih.gov/pubmed/11702832 (accessed 5 Jul 2018).

5 Kocken M, Uijterwaal MH, de Vries ALM, *et al.* High-risk human papillomavirus testing versus cytology in predicting post-treatment disease in women treated for high-grade cervical disease: a systematic review and meta-analysis. *Gynecol Oncol* 2012;**125**:500–7. doi:10.1016/j.ygyno.2012.01.015

6 Sellors JW, Sankaranarayanan R. Colposcopy and Treatment of Cervical Intraepithelial Neoplasia: A Beginners’ Manual. https://screening.iarc.fr/doc/Colposcopymanual.pdf (accessed 5 Jul 2018).

7 Whiting PF, Rutjes AWS, Westwood ME, *et al.* QUADAS-2: A Revised Tool for the Quality Assessment of Diagnostic Accuracy Studies. *Ann Intern Med* 2011;**155**:529. doi:10.7326/0003-4819-155-8-201110180-00009
