# Supplementary material for: Screening test accuracy of portable devices that can be used to perform colposcopy for detecting CIN2+ in low- and middle-income countries: a systematic review and meta-analysis
Source: BMC Womens Health. 2020 Nov 16;20:253. doi: 10.1186/s12905-020-01121-3 (PMC7670616; doi:10.1186/s12905-020-01121-3)
Supplement: Supplementary file 2 — Additional file 2. “Medline Ovid search strategy”. Description of the Medline Ovid search strategy. [file 12905_2020_1121_MOESM2_ESM.doc]

Database: Ovid MEDLINE Search Strategy:

--------------------------------------------------------------------------------

1 Uterine Cervical Diseases/di, dg, ep, et, pa, pp, pc, vi

2 Uterine Cervical Neoplasms/an, di, dg, ep, et, pa, pc, pp, vi

3 Uterine Cervical Dysplasia/an, di, dg, ep, et, pa, pc, pp, vi

4 Cervical Intraepithelial Neoplasia/di, dg, ep, et, pa, pp, pc, vi

5 "Squamous Intraepithelial Lesions of the Cervix"/di, ep, et, pa, pc, vi

6 "Atypical Squamous Cells of the Cervix"/cl, cy, pa, vi

7 Cervix Uteri/ab, an, di, dg, ep, et, pa, pp, vi

8 Carcinoma, Squamous Cell/an, di, dg, ep, et, pa, pc, th, vi

9 ((cervix or cervical or cervico*) and (cancer or cancerous or precancer* or pre-cancer* or premalignan* or carcinoma* or adenocarcinoma* or dysplas* or neoplas* or dyskaryos* or squamous or squamocolumnar or CIN or CIN1* or CINI* or CIN2* or CINII* or CIN3* or CINIII* or SIL or HSIL or H-SIL or LSIL or L-SIL or ASCUS or ASC-US or "ASC US" or ASC-R or lsil or ASC-H)).ti,ab,kw.

10 or/1-9

11 Mass Screening/ or "Early Detection of Cancer"/ or Vaginal Smears/ or Papanicolaou Test/ or Diagnostic Equipment/

12 (screening* or detect* or test or tests or testing* or tested or diagnos* or triag*).ti,ab,kw.

13 (vagina* adj5 smear*).ti,ab,kw.

14 (pap* adj5 (test* or smear*)).ti,ab,kw.

15 (cervi* adj5 (smear* or screen*)).ti,ab,kw.

16 ((cytology or cytobrush) and cervi*).ti,ab,kw.

17 or/11-16

18 Colposcopes/

19 Colposcopy/

20 exp Spectrum Analysis/

21 colposcop*.ti,ab,kw.

22 tomography.ti,ab,kw.

23 spectro*.ti,ab,kw.

24 reflectance*.ti,ab,kw.

25 fluorescence*.ti,ab,kw.

26 hyperspectr*.ti,ab,kw.

27 LuViva.ti,ab,kw.

28 telecolposcop*.ti,ab,kw.

29 multispectr*.ti,ab,kw.

30 dysis.ti,ab,kw.

31 spectra*.ti,ab,kw.

32 (optical adj3 colpo*).ti,ab,kw.

33 (advanced adj3 cervi* adj3 scan*).ti,ab,kw.

34 optical imag*.ti,ab,kw.

35 microcolpo*.ti,ab,kw.

36 point probe.ti,ab,kw.

37 confocal endo*.ti,ab,kw.

38 confocal microscop*.ti,ab,kw.

39 (computerized adj3 colpo*).ti,ab,kw.

40 (digital* or digitized).ti,ab,kw.

41 truscreen.ti,ab,kw.

42 ((Enhanced adj3 Visual adj3 Assessment) or EVA or mHealth or eHealth or mobile? or smartphone? or telemedicine or web-based).ti,ab,kw.

43 (Gynocular* or (magnifying adj3 device?)).ti,ab,kw.

44 or/18-43

45 Point-of-Care Systems/

46 (point-of-care or see-and-treat).ti,ab,kw.

47 (community adj3 health adj3 technol*).ti,ab,kw.

48 (ambulatory adj3 care adj3 facilit*).ti,ab,kw.

49 real-time.ti,ab,kw.

50 mobile$1.ti,ab,kw.

51 tablet$1.ti,ab,kw.

52 (iphone$1 or smartphone$1).ti,ab,kw.

53 (web-based or online).ti,ab,kw.

54 compact.ti,ab,kw.

55 portable.ti,ab,kw.

56 hand-held.ti,ab,kw.

57 pocket-size*.ti,ab,kw.

58 rechargeable.ti,ab,kw.

59 battery-driven.ti,ab,kw.

60 telemedicine.ti,ab,kw.

61 mHealth.ti,ab,kw.

62 computeri#ed.ti,ab,kw.

63 or/45-62

64 10 and 17 and 44 and 63

***************************
