# Supplementary material for: Screening test accuracy of portable devices that can be used to perform colposcopy for detecting CIN2+ in low- and middle-income countries: a systematic review and meta-analysis
Source: BMC Womens Health. 2020 Nov 16;20:253. doi: 10.1186/s12905-020-01121-3 (PMC7670616; doi:10.1186/s12905-020-01121-3)
Supplement: Supplementary file 3 — Additional file 3. “Full text assessments_explanation for exclusions”. Table showing excluded full texts and explanations for their exclusion. [file 12905_2020_1121_MOESM3_ESM.pdf]

Number	Authors	Title	Year	Reason for exclusion	Device details	Comments
1	Abdel-Hady, E. S., Fawzy, M., Mesbah, A., Abdel-Hady, R., Gamal, A., Barakat, R.	Early detection of cervical precancerous changes in low resource settings: Can the performance of visual inspection with acetic acid test be improved?	2014	*Irrelevent study type or subject*		
2	Abdul, S., Brown, B. H., Milnes, P., Tidy, J. A.	The use of electrical impedance spectroscopy in the detection of cervical intraepithelial neoplasia	2006	*Index test does not fit inclusion criteria*	Electrical impedance spectroscopy (EIS)	
3	Adelman, M. R.	Novel advancements in colposcopy: Historical perspectives and a systematic review of future developments	2014	*Irrelevent study type or subject*		
4	Adler, J. J., Bloss, C. M., Jr., Mosley, K. T.	The Oklahoma State Department of Health Mobile Multiphasic Screening Program for Chronic Disease. II	1966	*Irrelevent study type or subject*		
5	Aggarwal P1, Batra S, Gandhi G, Zutshi V.	Can visual inspection with acetic acid under magnification substitute colposcopy in detecting cervical intraepithelial neoplasia in low-resource settings?	2011	*Index test does not fit inclusion criteria*	Magnivisualizer	optical magnification 4X
6	Agnes Vijaya, U., Purushothama Reddy, K., Srinivasullu, M., Mallesh, M.	Truscreen - An optoelectronic device with "real-time results - A new paradigm in cervical cancer screening"	2015	*Index test does not fit inclusion criteria*	Truscreen	
7	Alexandrov, S. A., Uttam, S., Bista, R. K., Zhao, C., Liu, Y.	Real-time quantitative visualization of 3D structural information	2012	*Irrelevent study type or subject*		
8	Amin, K., Davis, M., Dillon, S., Danley, M., Diaz, F., Tawfik, L., Hernandez-Rios, P., Wilson, J., Fan, F., Madan, R., Tawfik, O.	Digitally assisted review of pap smear cellblock preparations "telepapology" is a valid screening/diagnostic method	2011	*Irrelevent study type or subject*		
9	An, R., He, D., Xue, Y., Wang, S., Xie, L., Zhao, J., Wang, X., Yang, L.	Real-time detection of survivin mRNA expression in cervical cancer cell lines using molecular beacon imaging	2006	*Index test does not fit inclusion criteria*	Other histology or cytology related devices	
10	Ascencio, M., Collinet, P., Cosson, M., Mordon, S.	The role and value of optical coherence tomography in gynecology	2007	*Index test does not fit inclusion criteria*	Other histology or cytology related devices	
11	Ascencio, M., Collinet, P., Cosson, M., Mordon, S.	The place of optical coherence tomography in gynecology	2007	*Index test does not fit inclusion criteria*	Other histology or cytology related devices	
12	Asgary, R., Adongo, P. B., Nwameme, A., Cole, H. V. S., Maya, E., Liu, M., Yeates, K., Adanu, R., Ogedegbe, O.	MHealth to Train Community Health Nurses in Visual Inspection with Acetic Acid for Cervical Cancer Screening in Ghana	2016	*Irrelevent study type or subject*		
13	Asiedu M.N., Simhal A., Chaudhary U., Mueller J.L., Lam C.T., Schmitt J.W., Venegas G., Sapiro G., Ramanujam N.	Development of Algorithms for Automated Detection of Cervical Pre-Cancers with a Low-Cost, Point-of-Care, Pocket Colposcope	2019	*Irrelevent study type or subject*		
14	Asiedu, M. N., Guillermo, S., Ramanujam, N.	Low-cost, speculum-free, automated cervical cancer screening: Bringing expert colposcopy assessment to community health	2017	*Test accuracy data not included*		
15	Atanasova, D., Zlatkov, V.	CLINICAL PERFORMANCE OF DIFFERENT METHODS FOR CERVICAL SCREENING	2015	*Index test does not fit inclusion criteria*	Truscreen	
16	Atanassova, D., Zlatkov, V., Borisov, S., Veleva, G.	[Diagnostic value of TruScreen, cytology and colposcopy]	2013	*Index test does not fit inclusion criteria*	Electrical impedance spectroscopy (EIS)	
17	Atkinson, E. N.	Age and FSH effects in fluorescence spectra from the cervix: An exploratory analysis	2005	*Index test does not fit inclusion criteria*	Electrical impedance spectroscopy (EIS)	
18	Atkinson, E. N., Mitchell, M. F., Ramanujam, N., Richards-Kortum, R.	Statistical techniques for diagnosing CIN using fluorescence spectroscopy: SVD and CART	1995	*Irrelevent study type or subject*		
19	Aue-Aungkul, A., Punyawatanasin, S., Natprathan, A., Srisomboon, J., Kietpeerakool, C.	"See and treat" approach is appropriate in women with high-grade lesions on either cervical cytology or colposcopy	2011	*Index test does not fit inclusion criteria*	Stationary colposcope	
20	Badaya, S., Singh, S.	Missing cervical cancer patients in India: Time to rejuvenate follow up strategy through mhealth	2015	*Irrelevent study type or subject*		
21	Badizadegan, K., Backman, V., Boone, C. W., Crum, C. P., Dasari, R. R., Georgakoudi, I., Keefe, K., Munger, K., Shapshay, S. M., Sheets, E. E., Feld, M. S.	Spectroscopic diagnosis and imaging of invisible pre-cancer	2004	*Irrelevent study type or subject*		
22	Bae, S. J., Lee, D. S., Berezin, V., Kang, U., Lee, K. H.	Multispectral autofluorescence imaging for detection of cervical lesions: A preclinical study	2016	*Irrelevent study type or subject*		
23	Balasubramani, L., Brown, B. H., Healey, J., Tidy, J. A.	The detection of cervical intraepithelial neoplasia by electrical impedance spectroscopy: The effects of acetic acid and tissue homogeneity	2009	*Index test does not fit inclusion criteria*	Electrical impedance spectroscopy (EIS)	
24	Banerjee, D., Shemer, E., Basu, P.	Diagnostic accuracy of a portable field colposcope in a via and HPV detection based cervical cancer screening program	2015	*Related to studies that are already included*		
25	Basta, A., Szczudrawa, A., Pitynski, K., Kolawa, W.	The value of colposcopy and computerised colposcopy in diagnosis and therapeutic management of CIN and early invasive cervical cancer in pregnant women	2002	*Index test does not fit inclusion criteria*	Stationary colposcope	
26	Basu, P., Banerjee, D., Mittal, S., Mandal, R., Ghosh, I., Das, P., Muwonge, R., Biswas, J.	Evaluation of a compact, rechargeable, magnifying device to triage VIA and HPV positive women in a cervical cancer screening program in rural India	2016	*Related to studies that are already included*		
27	Bazant-Hegemark, F., Edey, K., Swingler, G. R., Read, M. D., Stone, N.	Review: Optical micrometer resolution scanning for non-invasive grading of precancer in the human uterine cervix	2008	*Irrelevent study type or subject*		
28	Bessey, A., Whyte, S., Minton, J., Chilcott, J.	Economic evaluation of an electrical impedance spectroscopy (EIS) device used asan adjunct to colposcopy	2013	*Index test does not fit inclusion criteria*	Electrical impedance spectroscopy (EIS)	
29	Bishai, D. M., Ferris, D. G., Litaker, M. S.	What is the Least Costly Strategy to Evaluate Cervical Abnormalities in Rural Women? Comparing Telemedicine, Local Practitioners, and Expert Physicians	2003	*Irrelevent study type or subject*		
30	Bloch, B.	An economic appraisal of a mobile cervical cytology screening service	1997	*Irrelevent study type or subject*		
31	Bose, C. K., Basak, J., Basu, N., Mukhopadhyay, A.	Report of a CAMP approach cervical screening and management effort in eastern india	2014	*Irrelevent study type or subject*		
32	Cardenas-Turanzas, M., Freeberg, J. A., Benedet, J. L., Atkinson, E. N., Cox, D. D., Richards-Kortum, R., MacAulay, C., Follen, M., Cantor, S. B.	The clinical effectiveness of optical spectroscopy for the in vivo diagnosis of cervical intraepithelial neoplasia: Where are we?	2007	*Irrelevent study type or subject*		
33	Carlson, K., Chidley, M., Sung, K. B., Descour, M., Gillenwater, A., Follen, M., Richards-Kortum, R.	In vivo fiber-optic confocal reflectance microscope with an injection-molded plastic miniature objective lens	2005	*Index test does not fit inclusion criteria*	Confocal microscopy	
34	Castro, C. M., Im, H., Pathania, D., Weissleder, R., Lee, H.	Illuminating the shadows of cervical disease using a mobile digital diffraction platform	2016	*Irrelevent study type or subject*		
35	Catarino, R., Vassilakos, P., Scaringella, S., Undurraga-Malinverno, M., Meyer-Hamme, U., Ricard-Gauthier, D., Matute, J. C., Petignat, P.	Smartphone use for cervical cancer screening in low-resource countries: A pilot study conducted in Madagascar	2015	*Index test does not fit inclusion criteria*	VIA/VILI/Smartphone	2x optical zoom
36	Chang, S. K., Follen, M., Malpica, A., Utzinger, U., Staerkel, G., Cox, D., Atkinson, E. N., MacAulay, C., Richards-Kortum, R.	Optimal excitation wavelengths for discrimination of cervical neoplasia	2002	*Index test does not fit inclusion criteria*	Electrical impedance spectroscopy (EIS)	
37	Chang, S. K., Mirabal, Y. N., Atkinson, E. N., Cox, D., Malpica, A., Follen, M., Richards-Kortum, R.	Combined reflectance and fluorescence spectroscopy for in vivo detection of cervical pre-cancer	2005	*Index test does not fit inclusion criteria*	Electrical impedance spectroscopy (EIS)	
38	Chang, V. T., Merisier, D., Yu, B., Walmer, D. K., Ramanujam, N.	Towards a field-compatible optical spectroscopic device for cervical cancer screening in resource-limited settings: effects of calibration and pressure	2011	*Index test does not fit inclusion criteria*	Electrical impedance spectroscopy (EIS)	
39	Chaturvedi, P., Majumder, S. K., Krishna, H., Muttagi, S., Gupta, P. K.	Fluorescence spectroscopy for noninvasive early diagnosis of oral mucosal malignant and potentially malignant lesions	2010	*Irrelevent study type or subject*		
40	Chen, Z. P., Chen, H. M., Lee, T. T.	Use of compact digital cervicography: An adjuvant screening tool for precancerous cervical lesions	2008	*Index test does not fit inclusion criteria*	DC /VIAC	ref standard Pap Canon 350D digital camerawith a Canon EFS 60-mm macro lens and a circular-shaped continuous light source.
41	Chibwesha, C. J., Frett, B., Katundu, K., Bateman, A. C., Shibemba, A., Kapambwe, S., Mwanahamuntu, M. H., Banda, S., Hamusimbi, C., Polepole, P., Parham, G. P.	Clinical Performance Validation of 4 Point-of-Care Cervical Cancer Screening Tests in HIV-Infected Women in Zambia	2016	*Index test does not fit inclusion criteria*	DC /VIAC	Acetic acid only, no lugols no green filter, DC photographs captured with a commercial brand camera for magnification
42	Cholli P., Manga S., Bradford L.S., Ngonwei E., Manju F., Wamai R., DeGregorio G., Tih P.M., Sheldon L.K., Ogembo R., Liu Y., Nulah K., Welty E., Welty T., Ogembo J.G.	Implementation of carehpv screening followed by via-dc to triage treatment of cervical neoplasia and cancer in cameroon	2015	*Irrelevent study type or subject*	DC /VIAC abstract only	
43	Collier, T., Guillaud, M., Follen, M., Malpica, A., Richards-Kortum, R.	Real-time reflectance confocal microscopy: comparison of two-dimensional images and three-dimensional image stacks for detection of cervical precancer	2007	*Index test does not fit inclusion criteria*	Confocal microscopy	
44	Coppleson, M., Canfell, K., Skladnev, V.	The polarprobe - An instantaneous optoelectronic approach to cervical screening	2000	*Index test does not fit inclusion criteria*	Other histology or cytology related devices	
45	Cristoforoni, P. M., Gerbaldo, D., Perino, A., Piccoli, R., Montz, F. J., Capitanio, G. L.	Computerized colposcopy: Results of a pilot study and analysis of its clinical relevance	1995	*Index test does not fit inclusion criteria*	Stationary colposcope	
46	Degueldre, M., Thomassin, J., Vandromme, J., De Wind, A., Jauffret, C., Lambaudie, E.	Real time confocal endomicroscopy for the in vivo exploration of the cervix	2015	*Index test does not fit inclusion criteria*	Confocal microscopy	
47	Degueldre, M., Vandromme, J., De Wind, A., Feoli, F.	Real-time in-vivo microscopic imaging of the cervix using confocal laser endomicroscopy: Preliminary observations and feasibility study	2016	*Index test does not fit inclusion criteria*	Confocal microscopy	
48	Deligdisch, L.	[Optical biopsy in gynecology]	2011	*Index test does not fit inclusion criteria*	Other histology or cytology related devices	
49	Denkceken, T., Simsek, T., Erdogan, G., Pestereli, E., Karaveli, S., Ozel, D., Bilge, U., Canpolat, M.	Elastic light single-scattering spectroscopy for the detection of cervical precancerous ex vivo	2013	*Index test does not fit inclusion criteria*	Electrical impedance spectroscopy (EIS)	
50	Di Pierro, F., Di Paola, G., Risso, P.	Role of a medical device for intra-vaginal use in improving the quality of the colposcopic examination and the anatomical/pathological reading of the cytological test and biopsy	2014	*Index test does not fit inclusion criteria*	Stationary colposcope	
51	Dong Y.	The study of artificial intelligence and cloud computing in cervical cancer screening program in shanxi province, china	2018	*Index test does not fit inclusion criteria*	AI	
52	Drezek, R. A., Collier, T., Brookner, C. K., Malpica, A., Lotan, R., Richards-Kortum, R. R., Follen, M.	Laser scanning confocal microscopy of cervical tissue before and after application of acetic acid	2000	*Index test does not fit inclusion criteria*	Other histology or cytology related devices	
53	Ebisch, R. M. F., Hermens, M., Van Den Akker, P. A. J., Massuger, L. F. A. G., Melchers, W. J. G., Bekkers, R. L. M.	Multimodal Hyperspectroscopic Imaging for Detection of High-Grade Cervical Intraepithelial Neoplasia	2017	*Index test does not fit inclusion criteria*	Electrical impedance spectroscopy (EIS)	
54	Engelstad, L. P., Stewart, S. L., Nguyen, B. H., Bedeian, K. L., Rubin, M. M., Pasick, R. J., Hiatt, R. A.	Abnormal Pap smear follow-up in a high-risk population	2001	*Irrelevent study type or subject*		
55	Etherington, I. J.	Telecolposcopy - a feasibility study in primary care	2002	*Index test does not fit inclusion criteria*	Stationary colposcope	
56	Etherington, I. J., Dunn, J., Shafi, M. I., Smith, T., Luesley, D. M.	Video colpography: a new technique for secondary cervical screening	1997	*Index test does not fit inclusion criteria*	Stationary colposcope	
57	Etherington, J., Watts, A. D., Hughes, E., Lester, H. E.	The use of telemedicine in primary care for women with cervical cytological abnormalities	2002	*Index test does not fit inclusion criteria*	Stationary colposcope	
58	Ferris, D. G., Bishai, D. M., Litaker, M. S., Dickman, E. D., Miller, J. A., Macfee, M. S.	Telemedicine Network Telecolposcopy Compared with Computer-Based Telecolposcopy	2004	*Index test does not fit inclusion criteria*	Stationary colposcope	
59	Ferris, D. G., Shapiro, J., Fowler, C., Cutler, C., Waller, J., Condorhuaman, W. S. G.	The Impact of Accessible Cervical Cancer Screening in Peru - The Dia del Mercado Project	2015	*Irrelevent study type or subject*		
60	Firmino-Machado J., Varela S., Mendes R., Moreira A., Lunet N., Carmo A., Cancela A., Firmino A., Ramos A., Teixeira A., Vieira A., Badim B., Tojal C., Junqueira C., Pinheiro C., Peneda E., Monte H., Vieira H.M., Proenca I., Seabra J., Teixeira J., Magalhaes J., Batista J., Silva J., Grijo L., Beirao L., Castanheira M., Silva M., Peixoto M.J., Ponto Santos M., Neves M., Amaral M., Capela N., Santos P., Apolinario P., Aguiar R., Barbosa R., Amendoeira R., Medon R., Pinheiro Torres S., Silva S., Fernandes T., Santos V.	A 3-step intervention to improve adherence to cervical cancer screening: The SCAN randomized controlled trial	2019	*Irrelevent study type or subject*		

61	Firnhaber, C., Mao, L., Levin, S., Faesen, M., Lewis, D. A., Goeieman, B. J., Swarts, A. J., Rakhombe, N., Michelow, P. M., Williams, S., Smith, J. S.	Evaluation of a cervicography-based program to ensure quality of visual inspection of the cervix in HIV-infected women in Johannesburg, South Africa	2015	*Index test does not fit inclusion criteria*	DC /VIAC	Acetic acid only, PowerShot A590 IS 8-megapixel (3264 x 2448) camera (Canon, Chiba, Japan) with LA-DC52G lens adaptor (Canon) and 250D close-up lens digital camera (Canon)
62	Flowers, L. C., Tadros, T. S.	Multimodal spectroscopy as a triage test for women at risk for cervical neoplasia: Results for adolescent subjects	2010	*Index test does not fit inclusion criteria*	Electrical impedance spectroscopy (EIS)	
63	Fokom Domgue, J., Kikah, E., Nkene, Y., Tebeu, P. M.	Feasibility and utility of colposcopy for screening and management of cervical dysplasia in a tertiary level hospital in a low-income setting	2016	*Index test does not fit inclusion criteria*	Stationary colposcope	
64	Freeberg, J. A., Benedet, J. L., MacAulay, C., West, L. A., Follen, M.	The performance of fluorescence and reflectance spectroscopy for the in vivo diagnosis of cervical neoplasia; point probe versus multispectral approaches	2007	*Index test does not fit inclusion criteria*	Electrical impedance spectroscopy (EIS)	
65	Freeberg, J. A., Benedet, J. L., West, L. A., Atkinson, E. N., MacAulay, C., Follen, M.	The clinical effectiveness of fluorescence and reflectance spectroscopy for the in vivo diagnosis of cervical neoplasia: An analysis by phase of trial design	2007	*Index test does not fit inclusion criteria*	Electrical impedance spectroscopy (EIS)	
66	Gallay, C., Girardet, A., Viviano, M., Catarino, R., Benski, A. C., Tran, P. L., Ecabert, C., Thiran, J. P., Vassilakos, P., Petignat, P.	Cervical cancer screening in low-resource settings: A smartphone image application as an alternative to colposcopy	2017	*Index test does not fit inclusion criteria*	VIA/VILI/Smartphone	1.39 - 1.69 optical zoom
67	Gallwas, J., Chiapponi, C., Turk, L., Ochsenkuehn, R., Friese, K., Dannecker, C.	Optical coherence tomography: Preliminary results with a new noninvasive technique for evaluating uterine cervical tissue and vulvar epithelium	2010	*Index test does not fit inclusion criteria*	Other histology or cytology related devices	
68	Gallwas, J., Turk, L., Friese, K., Dannecker, C.	Optical coherence tomography as a non-invasive imaging technique for preinvasive and invasive neoplasia of the uterine cervix	2010	*Index test does not fit inclusion criteria*	Other histology or cytology related devices	
69	Gallwas, J., Turk, L., Stepp, H., Friese, K., Dannecker, C.	Visualization of cervical intraepithelial neoplasias by optical coherence tomography - Early results	2009	*Index test does not fit inclusion criteria*	Other histology or cytology related devices	
70	Gamboa O., Gonzalez M., Bonilla J., Luna J., Murillo R., Inc C.C.S.S.G.	Visual techniques for cervical cancer screening in Colombia	2019	*Index test does not fit inclusion criteria*	Stationary colposcope	
71	Gardeil, F., Turner, M. J.	The case for selective 'see and treat' in patients referred for colposcopy	1995	*Index test does not fit inclusion criteria*	Stationary colposcope	
72	Gegzna, V., Vaitkuvien, A., Kurtinaitien, R., Vaitkus, J.	Fluorescence of cervical smear material for photo diagnostics	2010	*Index test does not fit inclusion criteria*	Electrical impedance spectroscopy (EIS)	
73	Goldstein L., Goldstein A., Lipson R., He A.	Self-sampling HPV testing combined with digital cervicography in a see-and-treat cervical cancer screening program	2018	*Irrelevent study type or subject*		
74	Goldstein L., Goldstein A., Lipson R., He A.	Self-sampling HPV testing combined with digital cervicography in a see-and-treat cervical cancer screening program	2018	*Index test does not fit inclusion criteria*	EVA	optical magnification 4X
75	Goldstein L., Thay S., Goldstein A., Lim K., Seang C.	Determining the optimal cervical carcinoma screening method in HIV positive and HIV negative cambodian women	2018	*Irrelevent study type or subject*		
76	Goldstein, L., Kellogg-Spadt, S., Marfori, C., Goldstein, A.	Digital cervicography for quality control of visualization with acetic acid (via) for cervical dysplasia screening	2017	*Test accuracy data not included*		
77	Golubovic, M., Lopicic, M., Terzic, N., Djurovic, M., Mugosa, B., Mijovic, G.	Presence of histopathological premalignant lesions and infections caused by high-risk genotypes of human papillomavirus in patients with suspicious cytological and colposcopy results - A prospective study	2017	*Index test does not fit inclusion criteria*	Stationary colposcope	
78	Gonzalez-Solis, J. L., Martinez-Espinosa, J. C., Torres-Gonzalez, L. A., Aguilar-Lemarroy, A., Jave-Suarez, L. F., Palomares-Anda, P.	Cervical cancer detection based on serum sample Raman spectroscopy	2014	*Index test does not fit inclusion criteria*	Electrical impedance spectroscopy (EIS)	
79	Gormley, R. H., Quinley, K. E., Shih, T., Zsofia, Szep, Steiner, A., Ramogola-Masire, D., Kovarik, C. L.	Use of mobile telemedicine for cervical cancer screening of HIV-positive women in Gaborone, Botswana	2010	*Index test does not fit inclusion criteria*	VIA/VILI/Smartphone	protocol paper change reason for exclusion
80	Grant, B. D., Fregnani, J. H. T. G., Possati Resende, J. C., Scapulatempo-Neto, C., Matsushita, G. M., Mauad, E. C., Quang, T., Stoler, M. H., Castle, P. E., Schmeler, K. M., Richards-Kortum, R. R.	High-resolution microendoscopy: A point-of-care diagnostic for cervical dysplasia in low-resource settings	2017	*Index test does not fit inclusion criteria*	Confocal microscopy	
81	Grant, Benjamin D, Quang, Timothy, Possati-Resende, Julio Cesar, Scapulatempo-Neto, Cristovam, de Macedo Matsushita, Graziela, Mauad, Edmundo Carvalho, Stoler, Mark H, Castle, Philip E, Guerreiro Fregnani, Jose Humberto Tavares, Schmeler, Kathleen M, Richards-Kortum, Rebecca	A mobile-phone based high-resolution microendoscope to image cervical precancer.	2019	*Index test does not fit inclusion criteria*	Confocal microscopy	
82	Greenbaum, A., Sikora, U., Ozcan, A.	Field-portable wide-field microscopy of dense samples using multi-height pixel super-resolution based lensfree imaging	2012	*Index test does not fit inclusion criteria*	Confocal microscopy	
83	Greenbaum, A., Zhang, Y., Feizi, A., Chung, P. L., Luo, W., Kandukuri, S. R., Ozcan, A.	Wide-field computational imaging of pathology slides using lens-free on-chip microscopy	2014	*Index test does not fit inclusion criteria*	Confocal microscopy	
84	Guerra, R., Montealegre, J. R., Robazetti, S. C., Montealegre, A., Nugent, E., Lucci, J. A.	Innovative cancer screening model offering same-day testing and treatment	2016	*Index test does not fit inclusion criteria*	Stationary colposcope	
85	Guo, X., Abliz, G., Reyimu, H., Zhao, F., Kadeer, N., Matsidik, R., Wu, G., Abudula, A.	The association of a distinct plasma proteomic profile with the cervical high-grade squamous intraepithelial lesion of Uyghur women: A 2D liquid-phase chromatography/mass spectrometry study	2012	*Index test does not fit inclusion criteria*	Electrical impedance spectroscopy (EIS)	
86	Hariprasad, Roopa, Mehrotra, Ravi	Pocket colposcope: could it improve attendance and increase access to cervical cancer screening programmes?.	2018	*Irrelevent study type or subject*		
87	Hariri Tabrizi, S., Farzaneh, F., Aghamiri, S. M.	Applicability of optical reflectance spectroscopy for detection of precancerous lesions in uterine cervix in vivo	2014	*Index test does not fit inclusion criteria*	Electrical impedance spectroscopy (EIS)	
88	Hariri Tabrizi, S., Farzaneh, F., Aghamiri, S. M. R., Arab, M., Hosseini, M., Ashrafganjoei, T., Chehrazi, M.	Comparison between performance of single-fiber reflectance spectroscopy (SFRS) system and colposcopy: a phase III trial	2017	*Index test does not fit inclusion criteria*	Electrical impedance spectroscopy (EIS)	
89	Hartmann, K. E., Nanda, K., Hall, S., Myers, E.	Technologic advances for evaluation of cervical cytology: is newer better?	2001	*Irrelevent study type or subject*		
90	He, G., Lu, Y., Qian, X., Li, J., Yuan, Z., Li, C.	A coumarin-based fluorescence resonance energy transfer probe targeting matrix metalloproteinase-2 for the detection of cervical cancer	2017	*Index test does not fit inclusion criteria*	Truscreen	
91	He, X. K., Luo, X. P., Mao, L. Z., Chen, G. Y., Li, Y., Zhang, J. Y.	[An optoelectronic cervical cancer screening system for screening cervical cancer: comparison with cervical cytology]	2010	*Index test does not fit inclusion criteria*	Truscreen	
92	Hillemanns, P., Weingandt, H., Baumgartner, R., Diebold, J., Xiang, W., Stepp, H.	Photodetection of cervical intraepithelial neoplasia using 5- aminolevulinic acid-induced porphyrin fluorescence	2000	*Index test does not fit inclusion criteria*	Electrical impedance spectroscopy (EIS)	
93	Hitt, W. C., Low, G. M., Lynch, C. E., Gauss, C. H., Magann, E. F., Lowery, C. L., Eswaran, H.	Application of a Telecolposcopy Program in Rural Settings	2016	*Index test does not fit inclusion criteria*	Stationary colposcope	
94	Hitt, W. C., Low, G., Bird, T. M., Ott, R.	Telemedical cervical cancer screening to bridge medicaid service care gap for rural women	2013	*Index test does not fit inclusion criteria*	Stationary colposcope	
95	Ho, D., Drake, T. K., Smith-McCune, K. K., Darragh, T. M., Hwang, L. Y., Wax, A.	Feasibility of clinical detection of cervical dysplasia using angle-resolved low coherence interferometry measurements of depth-resolved nuclear morphology	2017	*Index test does not fit inclusion criteria*	Other histology or cytology related devices	
96	Hong, X., Nagarajan, V. K., Mugler, D. H., Yu, B.	Smartphone microendoscopy for high resolution fluorescence imaging	2016	*Index test does not fit inclusion criteria*	Confocal microscopy	
97	Hu, Liming, Bell, David, Antani, Sameer, Xue, Zhiyun, Yu, Kai, Horning, Matthew P, Gachuhi, Noni, Wilson, Benjamin, Jaiswal, Mayoore S, Befano, Brian, Long, L Rodney, Herrero, Rolando, Einstein, Mark H, Burk, Robert D, Demarco, Maria, Gage, Julia C, Rodriguez, Ana Cecilia, Wentzensen, Nicolas, Schiffman, Mark	An Observational Study of Deep Learning and Automated Evaluation of Cervical Images for Cancer Screening.	2019	*Index test does not fit inclusion criteria*	AI	
98	Huchko, M. J., Maloba, M., Cohen, C. R., Bukusi, E.	Performance of visual methods for cervical cancer screening among HIV-infected women in western kenya	2011	*Index test does not fit inclusion criteria*	VIA/VILI/smartphone	VIA/VILI
99	Hunt, Brady, Fregnani, Jose Humberto Tavares Guerreiro, Schwarz, Richard A, Pantano, Naitielle, Tesoni, Suelen, Possati-Resende, Julio Cesar, Antoniazzi, Marcio, de Oliveira Fonseca, Bruno, de Macedo Matsushita, Graziela, Scapulatempo-Neto, Cristovam, Kerr, Ligia, Castle, Philip E, Schmeler, Kathleen, Richards-Kortum, Rebecca	Diagnosing Cervical Neoplasia in Rural Brazil Using a Mobile Van Equipped with In Vivo Microscopy: A Cluster-Randomized Community Trial.	2018	*Index test does not fit inclusion criteria*	Confocal microscopy	
100	Im, H., Castro, C. M., Shao, H., Liong, M., Song, J., Pathania, D., Fexon, L., Min, C., Avila-Wallace, M., Zurkiya, O., Rho, J., Magaoay, B., Tambouret, R. H., Pivovarov, M., Weissleder, R., Lee, H.	Digital diffraction analysis enables low-cost molecular diagnostics on a smartphone	2015	*Irrelevent study type or subject*		
101	Kahn, B., Bernat, A., Cataldo, L., Bolton, F., Bar-Am, K., Jacques, S., Levitz, D.	Measuring blood content, oxygen saturation, and scattering in cervical tissues using multi-spectral imaging	2017	*Index test does not fit inclusion criteria*	Electrical impedance spectroscopy (EIS)	
102	Kahng, J., Kim, E. H., Kim, H. G., Lee, W.	Development of a cervical cancer progress prediction tool for human papillomavirus-positive Koreans: A support vector machine-based approach	2015	*Irrelevent study type or subject*		
103	Kanter, E. M., Vargis, E., Majumder, S., Keller, M. D., Woeste, E., Rao, G. G., Mahadevan-Jansen, A.	Application of Raman spectroscopy for cervical dysplasia diagnosis	2009	*Index test does not fit inclusion criteria*	Electrical impedance spectroscopy (EIS)	
104	Kim, S. N., Kim, Y. H., Nam, K. H., Lee, S. K., Lee, T. S., Choi, H. S., Han, S. J., Kim, S. C., Korean Cervicography Research, Group	Development and validation of novel digitalized cervicography system	2016	*Index test does not fit inclusion criteria*	Cervicscan	
105	Knapp, P.	The value of computerized topographic evaluation for CIN changes in the cervix of young women based on effectiveness of CO2 laser therapy	2000	*Index test does not fit inclusion criteria*	Electrical impedance spectroscopy (EIS)	
106	Knapp, P., Zbroch, T., Kobylec, M., Knapp, P.	[Computerized fluorocolposcopy--the significance in evaluation and therapy of early cervical lesions]	2004	*Index test does not fit inclusion criteria*	Electrical impedance spectroscopy (EIS)	
107	Krishnan, L., Bapat, A., Sakhilkar, R., Raje, S., Gaikwad, A., Busheri, L., Dixit, S., Koppiker, C. B.	Telemedicine-based community screening of cervical cancer	2017	*Test accuracy data not included*		
108	Kudva V., Prasad K., Guruvare S.	Andriod Device-Based Cervical Cancer Screening for Resource-Poor Settings	2018	*Index test does not fit inclusion criteria*	VIA/VILI/Smartphone	Android device AI development
109	Kumar, K., Hoshino, K., Zhang, X.	Handheld subcellular-resolution single-fiber confocal microscope using high-reflectivity two-axis vertical combdrive silicon microscanner	2008	*Index test does not fit inclusion criteria*	Confocal microscopy	
110	Lam, C. T., Krieger, M. S., Gallagher, J. E., Asma, B., Muasher, L. C., Schmitt, J. W., Ramanujam, N.	Design of a novel low cost point of care tampon (POCkeT) colposcope for use in resource limited settings	2015	*Irrelevent study type or subject*		
111	Lam, C. T., Mueller, J., Asma, B., Asiedu, M., Krieger, M. S., Chitalia, R., Dahl, D., Taylor, P., Schmitt, J. W., Ramanujam, N.	An integrated strategy for improving contrast, durability, and portability of a Pocket Colposcope for cervical cancer screening and diagnosis	2018	*Test accuracy data not included*		
112	Lasyk L., Gronwald J., Barbasz J., Zuk P., Prusaczyk A.J., Wlodarczyk T., Prokurat E., Olszewski W., Bidzinski M.	Evaluation of the constructed the device along with the software for digital archiving, sending the data and supporting the diagnosis of cervical cancer	2019	*Index test does not fit inclusion criteria*	AI	
113	le Roux, K., Prinsloo, L. C., Meyer, D.	Cellular injury evidenced by impedance technology and infrared microspectroscopy	2015	*Index test does not fit inclusion criteria*	Electrical impedance spectroscopy (EIS)	
114	Lee, J. S., Shuhatovich, O., Price, R., Pikkula, B., Follen, M., McKinnon, N., MacAulay, C., Knight, B., Richards-Kortum, R., Cox, D. D.	Design and preliminary analysis of a study to assess intra-device and inter-device variability of fluorescence spectroscopy instruments for detecting cervical neoplasia	2005	*Index test does not fit inclusion criteria*	Electrical impedance spectroscopy (EIS)	
115	Lee, K. H., Yong Seok, L.	Multispectral autofluorescence imaging for cervical cancer screening	2016	*Index test does not fit inclusion criteria*	Electrical impedance spectroscopy (EIS)	
116	Lee, S., Kim, J., Park, J.	A real time optoelectronic device and colposcopic Reid index in assessment of cervix	2009	*Index test does not fit inclusion criteria*	Truscreen	
117	Li, W., Soto-Thompson, M., Gustafsson, U.	A new image calibration system in digital colposcopy	2006	*Irrelevent study type or subject*		
118	Liu, Z., Belinson, S. E., Li, J., Yang, B., Wulan, N., Tresser, N. J., Wang, C., Mohr, M., Zhang, L., Zhou, Y., Weng, L., Wu, R., Belinson, J. L.	Diagnostic efficacy of real-time optical coherence tomography in the management of preinvasive and invasive neoplasia of the uterine cervix	2010	*Index test does not fit inclusion criteria*	Other histology or cytology related devices	
119	Lombardi, T. M., Kahn, B. S., Contreras, S., Waalen, J., Levitz, D.	Image comparison of a mobile colposcope (EVA) versus a standard colposcope for directing cervical biopsies in women with abnormal pap smears: A non-inferiority trial	2016	*Index test does not fit inclusion criteria*	EVA	optical magnification 4X
120	Long, S., Lei, W., Feng, Y., Lv, D., Cai, Y., Yang, P.	The feasibilities of TruScreen for primary cervical cancer screening: A self-controlled study	2013	*Index test does not fit inclusion criteria*	Truscreen	
121	Lua, R., Villanueva, B., Seanez, J., Ramirez, J., Suro, C.	Comparison of an optoelectronic scan of the cervix, cervical cytology and HPV-genotyping for cin screening	2017	*Index test does not fit inclusion criteria*	Truscreen	

122	Lynch, H. T., Harlan, W., Swartz, M., Marley, J., Becker, W., Lynch, J., Kraft, C., Krush, A. J.	Multiphasic mobile cancer screening: a positive approach to early cancer detection and control	1972	*Irrelevent study type or subject*		
123	Mahadevan-Jansen, A., Mitchell, M. F., Ramanujam, N., Utzinger, U., Richards-Kortum, R.	Development of a fiber optic probe to measure NIR Raman spectra of cervical tissue in vivo	1998	*Index test does not fit inclusion criteria*	Electrical impedance spectroscopy (EIS)	
124	Matti R., D'Sa D.K., Gupta V., Sebag C., Levitz D.	Comparison of visual and cytology cervical cancer screening in Maharashtra, India	2018	*Index test does not fit inclusion criteria*	EVA	optical magnification 4X
125	Maza, M., Schocken, C. M., Bergman, K. L., Randall, T. C., Cremer, M. L.	Cervical Precancer Treatment in Low- and Middle-Income Countries: A Technology Overview	2017	*Irrelevent study type or subject*		
126	Meena, B. L., Singh, P., Sah, A. N., Pandey, K., Agarwal, A., Pantola, C., Pradhan, A.	Intrinsic fluorescence for cervical precancer detection using polarized light based in-house fabricated portable device	2018	*Index test does not fit inclusion criteria*	Electrical impedance spectroscopy (EIS)	
127	Mikhail, M. S., Anyaegbunam, A., Romney, S. L.	Computerized colposcopy and conservative management of cervical intraepithelial neoplasia in pregnancy	1995	*Irrelevent study type or subject*		
128	Mikhail, M. S., Merkatz, I. R., Romney, S. L.	Clinical usefulness of computerized colposcopy: Image analysis and conservative management of mild dysplasia	1992	*Irrelevent study type or subject*		
129	Mink, J., Peterson, C.	MobileODT: a case study of a novel approach to an mHealth-based model of sustainable impact	2016	*Irrelevent study type or subject*		
130	Mink, J., Peterson, C., Pader, L., Rosenberg, Y., Levitz, D.	Statistics on clinical activities and practices of cervical cancer clinics in Kenya, and beyond	2017	*Index test does not fit inclusion criteria*	EVA	optical magnification 4X
131	Mirabal, Y. N., Chang, S. K., Atkinson, E. N., Malpica, A., Follen, M., Richards-Kortum, R.	Reflectance spectroscopy for in vivo detection of cervical precancer	2002	*Index test does not fit inclusion criteria*	Electrical impedance spectroscopy (EIS)	
132	Mitchell, M. F., Cantor, S. B., Ramanujam, N., Tortolero-Luna, G., Richards-Kortum, R.	Fluorescence spectroscopy for diagnosis of squamous intraepithelial lesions of the cervix	1999	*Index test does not fit inclusion criteria*	Electrical impedance spectroscopy (EIS)	
133	Mitchell, M. F., Hittelman, W. N., Lotan, R., Nishioka, K., Tortolero-Luna, G., Richards-Kortum, R., Hong, W. K.	Chemoprevention trials in the cervix: Design, feasibility, and recruitment	1995	*Index test does not fit inclusion criteria*	Electrical impedance spectroscopy (EIS)	
134	Mitra, A., Kalliala, I., Tzafetas, M., Bodai, Z., Rosini, F., Phelps, D., Gildea, L., Savage, A., Flora, R., Ghaem-Maghami, S., Takats, Z., Kyrgiou, M.	Use of rapid evaporative ionization mass spectrometry (REIMS) in cervical pathology-a proof of principle study	2017	*Index test does not fit inclusion criteria*	Electrical impedance spectroscopy (EIS)	
135	Moodley, J., Botha, H., Constant, D., Merwe, H. D., Momberg, M., Daskilewicz, K.	Exploring the feasibility of mobile technology to improve the management of clients with cervical cancer precursor lesions	2016	*Irrelevent study type or subject*		
136	Morens, A., Krief, B., Brugal, G.	The HOME microscope workstation. A new tool for cervical cancer screening	1992	*Index test does not fit inclusion criteria*	MICROSCOPE	
137	Mourant, J. R., Bocklage, T. J., Powers, T. M., Greene, H. M., Bullock, K. L., Marr-Lyon, L. R., Dorin, M. H., Waxman, A. G., Zsemlye, M. M., Smith, H. O.	In vivo light scattering measurements for detection of precancerous conditions of the cervix	2007	*Index test does not fit inclusion criteria*	Electrical impedance spectroscopy (EIS)	
138	Mourant, J. R., Bocklage, T. J., Powers, T. M., Greene, H. M., Dorin, M. H., Waxman, A. G., Zsemlye, M. M., Smith, H. O.	Detection of cervical intraepithelial neoplasias and cancers in cervical tissue by in vivo light scattering	2009	*Index test does not fit inclusion criteria*	Electrical impedance spectroscopy (EIS)	
139	Mueller, J. L., Asma, E., Lam, C. T., Krieger, M. S., Gallagher, J. E., Erkanli, A., Hariprasad, R., Malliga, J. S., Muasher, L. C., McHome, B., Oneko, O., Taylor, P., Venegas, G., Wanyoro, A., Mehrotra, R., Schmitt, J. W., Ramanujam, N.	International Image Concordance Study to Compare a Point-of-Care Tampon Colposcope with a Standard-of-Care Colposcope	2017	*Test accuracy data not included*		
140	Munoz-Zuluaga C., Sardi A., Orozco-Urdaneta M., Parra-Lara L.G., Perez A., El-Sharkawy F., Velez-Mejia C., King M.C.	Amate: A mobile application to improve access to early breast and cervical cancer detection	2018	*Irrelevent study type or subject*		
141	Murenzi G, Dusingize JC, Rurangwa T, Sinayobye JD, Munyaneza A, Murangwa A1, Zawadi T, Hebert T, Mugenzi P, Adedimeji A, Mutesa L, Anastos K, Castle PE.	Protocol for the study of cervical cancer screening technologies in HIV-infected women living in Rwanda.	2018	*Irrelevent study type or subject*		
142	Mwanahamuntu, M. H., Sahasrabuddhe, V. V., Blevins, M., Kapambwe, S., Shepherd, B. E., Chibwesha, C., Pfaendler, K. S., Mkumba, G., Vwalika, B., Hicks, M. L., Vermund, S. H., Stringer, J. S. A., Parham, G. P.	Utilization of Cervical Cancer Screening Services and Trends in Screening Positivity Rates in a 'Screen-And-Treat' Program Integrated with HIV/AIDS Care in Zambia	2013	*Irrelevent study type or subject*		
143	Nakappan, S., Park, S. Y., Serachitopol, D., Price, R., Cardeno, M., Au, S., Mackinnon, N., MacAulay, C., Follen, M., Pikkula, B. M.	Methodology of a real-time quality control for the multispectral digital colposcope (MDC)	2007	*Index test does not fit inclusion criteria*	Electrical impedance spectroscopy (EIS)	
144	Natarajan J., Vashist S., Mathur S., Tanwar P., Kumar S., Kriplani A., Singhal S., Meena J., Shemer E.W., Ramanujam N., Bhatla N.	A crossover randomized study of portable transvaginal colposcope and hand-held colposcope for evaluation of cervical intraepithelial neoplasia	2018	*Irrelevent study type or subject*		
145	Nessa, A., Roy, J. S., Chowdhury, M. A., Khanam, Q., Wistrand, C., Thuresson, M., Thorsell, M., Shemer, I., Wikstrom, S., Elisabeth, A.	Evaluation of the cervical swede score method and the gynocular by colposcopy trained VIA nurses: A cross-over randomised trial	2014	*Related to studies that are already included*		
146	Nessa, A., Wistrand, C., Begum, S. A., Thuresson, M., Shemer, I., Thorsell, M., Shemer, E. A. W.	Evaluation of stationary colposcope and the Gynocular, by the Swede score systematic colposcopic system in VIA positive women: A crossover randomized trial	2014	*Related to studies that are already included*		
147	Newman H., Jilin H., Zhu B., Bradford L., Gao G.	Evaluation of portable colposcopy and HPV testing for screening of cervical cancer in rural China	2019	*Related to studies that are already included*		
148	Ngonzi, J., Bajunirwe, F., Wistrand, C., Mayanja, R., Altman, D., Thorsell, M., Shemer, E. A. W.	Agreement of colposcope and Gynocular in assessment of cervical lesions by Swede score: A randomized, crossover pilot trial	2013	*Test accuracy data not included*		
149	Niu, J. J., Schrlau, M. G., Friedman, G., Gogotsi, Y.	Carbon nanotube-tipped endoscope for in situ intracellular surface-enhanced Raman spectroscopy	2011	*Index test does not fit inclusion criteria*	Electrical impedance spectroscopy (EIS)	
150	Ntziachristos, V.	Clinical translation of optical and optoacoustic imaging	2011	*Index test does not fit inclusion criteria*	Electrical impedance spectroscopy (EIS)	
151	Obahiagbon U., Smith J.T., Zhu M., Katchman B.A., Arafa H., Anderson K.S., Blain Christen J.M.	A compact, low-cost, quantitative and multiplexed fluorescence detection platform for point-of-care applications	2018	*Index test does not fit inclusion criteria*	Electrical impedance spectroscopy (EIS)	
152	Obahiagbon, Uwadiae, Smith, Joseph T, Zhu, Meilin, Katchman, Benjamin A, Arafa, Hany, Anderson, Karen S, Blain Christen, Jennifer M	A compact, low-cost, quantitative and multiplexed fluorescence detection platform for point-of-care applications.	2018	*Index test does not fit inclusion criteria*	Electrical impedance spectroscopy (EIS)	
153	Olaru, O. G., Stanescu, A. D., Nanu, D.	Development perspectives of screening and diagnosis methods of neoplastic diseases of the cervix	2013	*Index test does not fit inclusion criteria*	Electrical impedance spectroscopy (EIS)	
154	Orfanoudaki, I. M., Kappou, D., Sifakis, S.	Recent advances in optical imaging for cervical cancer detection	2011	*Irrelevent study type or subject*		
155	Orfanoudaki, I. M., Themelis, G. C., Sifakis, S. K., Fragouli, D. H., Panayiotides, J. G., Vazgiouraki, E. M., Koumantakis, E. E.	A clinical study of optical biopsy of the uterine cervix using a multispectral imaging system	2005	*Index test does not fit inclusion criteria*	Electrical impedance spectroscopy (EIS)	
156	Ozgu, E., Yildiz, Y., Ozgu, B. S., Oz, M., Danisman, N., Gungor, T.	Efficacy of a real time optoelectronic device (TruscreenTM) in detecting cervical intraepithelial pathologies: A prospective observational study	2015	*Index test does not fit inclusion criteria*	Truscreen	
157	Panchangam, A., Sastry, K. V., Rao, D. V., DeCristofano, B. S., Kimball, B. R., Nakashima, M.	Processing of medical images using real-time optical Fourier processing	2001	*Irrelevent study type or subject*		
158	Parashari, A., Singh, V., Sehgal, A., Mehrotra, R.	AV Magnivisualizer: A low-cost screening technology for early detection of precancerous and early cancerous lesions of the uterine cervix	2015	*Index test does not fit inclusion criteria*	Magnivisualizer	optical magnification 4X
159	Parham, G. P., Mwanahamuntu, M. H., Pfaendler, K. S., Sahasrabuddhe, V. V., Myung, D., Mkumba, G., Kapambwe, S., Mwanza, B., Chibwesha, C., Hicks, M. L., Stringer, J. S. A.	EC3-A modern telecommunications matrix for cervical cancer prevention in Zambia	2010	*Irrelevent study type or subject*		
160	Parra S., Keahey P., Richards-Kortum R.R., Schmeler K., Maza M., Castle P.	Development of a single-board computer high-resolution microendoscope (PiHRME) to detect cervical cancer in low-resource settings	2016	*Index test does not fit inclusion criteria*	Confocal microscopy	
161	Peron, Mathilde, Llewellyn, Alexis, Moe-Byrne, Thirimon, Walker, Simon, Walton, Matthew, Harden, Melissa, Palmer, Stephen, Simmonds, Mark	Adjunctive colposcopy technologies for assessing suspected cervical abnormalities: systematic reviews and economic evaluation.	2018	*Irrelevent study type or subject*		
162	Peterson, C. W., Rose, D., Mink, J., Levitz, D.	Real-time monitoring and evaluation of a visual-based cervical cancer screening program using a decision support job aid	2016	*Irrelevent study type or subject*		
163	Pierce, M. C., Guan, Y., Quinn, M. K., Zhang, X., Zhang, W. H., Qiao, Y. L., Castle, P., Richards-Kortum, R.	A pilot study of low-cost, high-resolution microendoscopy as a tool for identifying women with cervical precancer	2012	*Index test does not fit inclusion criteria*	Confocal microscopy	
164	Piyawattanametha, W.	Multi-Spectral Confocal Imaging Probe for cervical cancer imaging	2016	*Index test does not fit inclusion criteria*	Confocal microscopy	
165	Pogue, B. W., Mycek, M. A., Harper, D.	Image analysis for discrimination of cervical neoplasia	2000	*Index test does not fit inclusion criteria*	Stationary colposcope	
166	Prabitha, V. G., Suchetha, S., Jayanthi, J. L., Baiju, K. V., Rema, P., Anuraj, K., Mathews, A., Sebastian, P., Subhash, N.	Detection of cervical lesions by multivariate analysis of diffuse reflectance spectra: a clinical study	2016	*Index test does not fit inclusion criteria*	Electrical impedance spectroscopy (EIS)	
167	Prieto-Egido, I., Gonzalez-Escalada, A., Garcia-Giganto, V., Martinez-Fernandez, A.	Design of New Procedures for Diagnosing Prevalent Diseases Using a Low-Cost Telemicroscopy System	2016	*Index test does not fit inclusion criteria*	Confocal microscopy	
168	Pruski, D., Kedzia, W., Przybylski, M., Kedzia, H., Jozefiak, A., Spaczynski, M.	The assessment of a real - Time optoelectronic method for the detection of cervical intraepithelial neoplasia ('CIN')	2009	*Index test does not fit inclusion criteria*	Truscreen	
169	Quercia, K., Tran, P. L., Jinoro, J., Herniainasolo, J. L., Viviano, M., Vassilakos, P., Benski, C., Petignat, P.	A Mobile Health Data Collection System for Remote Areas to Monitor Women Participating in a Cervical Cancer Screening Campaign	2017	*Index test does not fit inclusion criteria*	Stationary colposcope	
170	Quinley, K. E., Gormley, R. H., Ratcliffe, S. J., Shih, T., Szep, Z., Steiner, A., Ramogola-Masire, D., Kovarik, C. L.	Use of mobile telemedicine for cervical cancer screening	2011	*Index test does not fit inclusion criteria*	VIA/VILI/Smartphone	Samsung SGH-U900
171	Quinn, M. K., Bubi, T. C., Pierce, M. C., Kayembe, M. K., Ramogola-Masire, D., Richards-Kortum, R.	High-Resolution Microendoscopy for the Detection of Cervical Neoplasia in Low-Resource Settings	2012	*Index test does not fit inclusion criteria*	Confocal microscopy	
172	Ramanujam, N., Mitchell, M. F., Mahadevan-Jansen, A., Thomsen, S. L., Staerkel, G., Malpica, A., Wright, T., Atkinson, N., Richards-Kortum, R.	Cervical precancer detection using a multivariate statistical algorithm based on laser-induced fluorescence spectra at multiple excitation wavelengths	1996	*Index test does not fit inclusion criteria*	Electrical impedance spectroscopy (EIS)	
173	Ramogola-Masire, D., de Klerk, R., Monare, B., Ratshaa, B., Friedman, H. M., Zetola, N. M.	Cervical cancer prevention in HIV-infected women using the "see and treat" approach in Botswana	2012	*Index test does not fit inclusion criteria*	DC /VIAC	VIA only EDI with a digital camera no further description on magnification capacity
174	Ratnavelu, N., Biliatis, I., Cross, P. A., Naik, R.	Ten-year outcomes of a one-stop colposcopy clinic: A unique service for low grade cytology	2013	*Index test does not fit inclusion criteria*	Stationary colposcope	
175	Ren, W., Qu, Y., Pei, J., Xiao, L., Zhang, S., Chang, S., Xu, R. X.	Development of a multimodal colposcopy for characterization of cervical intraepithelial neoplasia	2017	*Index test does not fit inclusion criteria*	Electrical impedance spectroscopy (EIS)	
176	Ricard-Gauthier, D., Wisniak, A., Catarino, R., Van Rossum, A. F., Meyer-Hamme, U., Negulescu, R., Scaringella, S., Jinoro, J., Vassilakos, P., Petignat, P.	Use of Smartphones as Adjuvant Tools for Cervical Cancer Screening in Low-Resource Settings	2015	*Index test does not fit inclusion criteria*	VIA/VILI/Smartphone	2X optical zoom Samsung Galaxy S4, Samsung Electronics, 2013, Seoul, South Korea)
177	Robichaux-Viehoever, A., Kanter, E., Shappell, H., Billheimer, D., Jones, Iii H., Mahadevan-Jansen, A.	Characterization of Raman spectra measured in vivo for the detection of cervical dysplasia	2007	*Index test does not fit inclusion criteria*	Electrical impedance spectroscopy (EIS)	
178	Rogalla, S., Contag, C. H.	Early cancer detection at the epithelial surface	2015	*Irrelevent study type or subject*		
179	Ronco, G., Caprioglio, A., Patriarca, S., Maina, G., Preti, M.	Risk of invasive cervical cancer after colposcopy with and without biopsy	2017	*Index test does not fit inclusion criteria*	Stationary colposcope	
180	Schadel, D., Coumbos, A., Ey, S., Willrodt, R. G., Albrecht, H., Kuhn, W.	Evaluation of a digital store-and-forward colposcopic system--a pilot study to assess usability for telemedicine	2005	*Index test does not fit inclusion criteria*	Stationary colposcope	
181	Schadel, D., Coumbos, A., Willrodt, R. G., Roggan, A., Jochum, T., Muller, G., Albrecht, H., Kuhn, W.	The role of digital colposcopy for diagnosis of cervical lesions - A pilot study to assess the value of telematics	2004	*Index test does not fit inclusion criteria*	Digital colp	not portable
182	Schmeler, K. M., Fregnani, J. T. G., Grant, B., Resende, J. P., Scapulatempo-Neto, C., Macedo, G., Stoler, M. H., Castle, P. E., Richards-Kortum, R.	High-resolution microendoscopy (HRME): A low-cost, point-of-care alternative to colposcopy and biopsies?	2015	*Index test does not fit inclusion criteria*	Confocal microscopy	
183	Mueller JL, Lam CT, Kellish M, Peters J, Asiedu MN, Krieger MS, Gallagher JE, Erkanli, A Ortiz E, Muasher LC, Taylor PT, Mchome BL, Oneko O, Quintero Venegas, Schmitt JW, Ramanujam N	Comparison of contrast mediators on the accuracy of a pocket colposcope in U.S., PERU, and sub-saharan Africa	2018	*Related to studies that are already included*	Only procedure done in Peru qualifies for inclusion, and more indepth data in another paper using same cohort	
184	Schneider, A., Zahm, D. M.	New adjunctive methods for cervical cancer screening	1996	*Index test does not fit inclusion criteria*	Truscreen	
185	Shah, H., Noronha, S.	Role of technology in cervical cancer diagnosis	2016	*Index test does not fit inclusion criteria*	Confocal microscopy	
186	Sharma, Drishti, Rohilla, Latika, Bagga, Rashmi, Srinivasan, Radhika, Jindal, Har Ashish, Sharma, Nikita, Kankaria, Ankita, Jamir, Limalemla, Suri, Vanita, Singh, Rakesh Kumar, Duggal, Mona	Feasibility of implementing cervical cancer screening program using smartphone imaging as a training aid for nurses in rural India.	2018	*Index test does not fit inclusion criteria*	VIA/VILI/Smartphone	OPTICAL MAG OR PHONE USED NOT STATED.
187	Shroff R., Shroff N., Thakur Y., Thakur V., Penketh R., Tas B.	An ultra low-cost, high-tech solution to expand the WHO recommended 'screen and treat' approach to address cervical cancer deaths in low and medium income countries	2018	*Irrelevent study type or subject*		

188	Singer, A., Coppleson, M., Canfell, K., Skladnev, V., Mackellar, G., Pisal, N., Deery, A.	A real time optoelectronic device as an adjunct to the Pap smear for cervical screening: A multicenter evaluation	2003	*Index test does not fit inclusion criteria*	Truscreen	
189	Singer, A., Shemer, E., Jordon, J.	New methods of teaching colposcopy	2017	*Irrelevent study type or subject*		
190	Singh, V., Parashari, A., Gupta, S., Sodhani, P., Sehgal, A.	Performance of a low cost magnifying device, magnivisualizer, versus colposcope for detection of pre-cancer and cancerous lesions of uterine cervix	2014	*Index test does not fit inclusion criteria*	Magnivisualizer	optical magnification 4X
191	Singhakum, Nissana, Laiwejpithaya, Somsak, Chaopotong, Pattama	Digital Cervicography by Simply Portable Device as an Alternative Test for Cervical Cancer Screening in Rural Area of Thailand.	2018	*Index test does not fit inclusion criteria*	DC /VIAC	VIA onlyUSB-pen camera agnification (4-15 times)
192	Smith, M. C., Broadhead, T. J., Hammond, R. H.	"See and treat" at colposcopy - Achieving the standard	2001	*Index test does not fit inclusion criteria*	Stationary colposcope	
193	Sokolov, K., Nieman, L. T., Myakov, A., Gillenwater, A.	Polarized Reflectance Spectroscopy for Pre-Cancer Detection	2004	*Index test does not fit inclusion criteria*	Electrical impedance spectroscopy (EIS)	
194	Svanberg, K.	Diagnostics and treatment of tumours using laser techniques	2010	*Index test does not fit inclusion criteria*	Electrical impedance spectroscopy (EIS)	
195	Svanberg, K.	Applications of laser spectroscopy to meet challenges in medicine	2013	*Index test does not fit inclusion criteria*	Electrical impedance spectroscopy (EIS)	
196	Swaddiwudhipong, W., Chaovakiratipong, C., Nguntra, P., Mahasakpan, P., Lerdlukanavonge, P., Koonchote, S.	Effect of a mobile unit on changes in knowledge and use of cervical cancer screening among rural Thai women	1995	*Index test does not fit inclusion criteria*	Stationary colposcope	
197	Swaddiwudhipong, W., Chaovakiratipong, C., Nguntra, P., Mahasakpan, P., Tatip, Y., Boonmak, C.	A mobile unit: An effective service for cervical cancer screening among rural Thai women	1999	*Irrelevent study type or subject*		
198	Taghavi, K., Banerjee, D., Mandal, R., Kopp Kallner, H., Thorsell, M., Friis, T., Kocoska-Maras, L., Strander, B., Singer, A., Shemer, E.	Colposcopy telemedicine: Live swede score versus static swede score and accuracy in detecting CIN2+	2017	*Related to studies that are already included*		
199	Tan, J. H. J., Wrede, C. D. H.	New technologies and advances in colposcopic assessment	2011	*Irrelevent study type or subject*		
200	Tan, J., Delaney, P., McLaren, W. J.	Confocal endomicroscopy: A novel imaging technique for in vivo histology of cervical intraepithelial neoplasia	2007	*Index test does not fit inclusion criteria*	Confocal microscopy	
201	Tan, J., Quinn, M. A., Pyman, J. M., Delaney, P. M., McLaren, W. J.	Detection of cervical intraepithelial neoplasia in vivo using confocal endomicroscopy	2009	*Index test does not fit inclusion criteria*	Confocal microscopy	
202	Tanaka, Y., Ueda, Y., Okazawa, A., Kakuda, M., Matsuzaki, S., Kobayashi, E., Yoshino, K., Kimura, T.	'Smartscopy' as an alternative device for cervical cancer screening: A pilot study	2017	*Index test does not fit inclusion criteria*	VIA/VILI/Smartphone	Apple iPhone 5S Specifications optical mag 1X
203	Tanaka, Yusuke, Ueda, Yutaka, Kakubari, Reisa, Kakuda, Mamoru, Kubota, Satoshi, Matsuzaki, Satoko, Okazawa, Akiko, Egawa-Takata, Tomomi, Matsuzaki, Shinya, Kobayashi, Eiji, Kimura, Tadashi	Histologic correlation between smartphone and coloposcopic findings in patients with abnormal cervical cytology: experiences in a tertiary referral hospital.	2019	*Index test does not fit inclusion criteria*	VIA/VILI/Smartphone	Apple iPhone 5S Specifications optical mag 1X
204	Thomas, A., Kummel, S., Gemeinhardt, O., Fischer, T.	Real-Time Sonoelastography of the Cervix: Tissue Elasticity of the Normal and Abnormal Cervix	2007	*Index test does not fit inclusion criteria*	Other histology or cytology related devices	
205	Tidy, J. A., Brown, B.	Improved colposcopic performance using electrical impedance spectroscopy (APX100) as an adjunct	2012	*Index test does not fit inclusion criteria*	Electrical impedance spectroscopy (EIS)	
206	Tidy, J., Brown, B., Healey, J., Daayana, S., Martin, M., Prendiville, W., Kitchener, H.	Improved detection of high-grade CIN using a hand held electrical spectrosopy device	2012	*Index test does not fit inclusion criteria*	Electrical impedance spectroscopy (EIS)	
207	Tidy, J., Healey, J., Brown, B.	Accuracy of detection of high-grade cervical intra-epithelial neoplasia using electrical impedance spectroscopy with colposcopy	2013	*Index test does not fit inclusion criteria*	Electrical impedance spectroscopy (EIS)	
208	Tran P.L., Benski C., Viviano M., Petignat P., Combescure C., Jinoro J., Herinianasolo J.L., Vassilakos P.	Performance of smartphone-based digital images for cervical cancer screening in a low-resource context	2018	*Index test does not fit inclusion criteria*	VIA/VILI/Smartphone	Samsung Galaxy S4 or S5, Seoul, South Korea 3.3–3.8× optical zoom same phone as above but states that only low mag used
209	Tumer, K., Ramanujam, N., Ghosh, J., Richards-Kortum, R.	Ensembles of radial basis function networks for spectroscopic detection of cervical precancer	1998	*Index test does not fit inclusion criteria*	Electrical impedance spectroscopy (EIS)	
210	Twiggs, L. B., Chakhtoura, N. A., Ferris, D. G., Flowers, L. C., Winter, M. L., Sternfeld, D. R., Lashgari, M., Burnett, A. F., Raab, S. S., Wilkinson, E. J.	Multimodal hyperspectroscopy as a triage test for cervical neoplasia: Pivotal clinical trial results	2013	*Index test does not fit inclusion criteria*	Electrical impedance spectroscopy (EIS)	
211	Twiggs, L. B., Chakhtoura, N. A., Werner, C. L., Griffith, W. F., Flowers, L. C., Lashgari, M., Ferris, D. G., Winter, M. L., Sternfeld, D. R., Burnett, A. F., Wilkinson, E. J., Raab, S. S.	Multimodal spectroscopy as a triage test for women at risk for cervical neoplasia: Results of a 1,607 subject pivotal trial	2010	*Index test does not fit inclusion criteria*	Electrical impedance spectroscopy (EIS)	
212	Urner, E., Delavy, M., Catarino, R., Viviano, M., Meyer-Hamme, U., Benski, A. C., Jinoro, J., Heriniainasolo, J. L., Undurraga, M., De Vuyst, H., Combescure, C., Vassilakos, P., Petignat, P.	A Smartphone-Based Approach for Triage of Human Papillomavirus-Positive Sub-Saharan African Women: A Prospective Study	2017	*Index test does not fit inclusion criteria*	VIA/VILI/Smartphone	Samsung Galaxy S4 and S5, 3.3-3.8× optical
213	Vaitkuviene, A., Ciplys, I., Varanius, D., Gegzna, V., Kurtinaitiene, R., Vaitkus, J. V.	Gender and age related peculiarities of fluorescence in medical samples	2017	*Index test does not fit inclusion criteria*	Electrical impedance spectroscopy (EIS)	zoom and in flash mode.
214	Vargis, E., Byrd, T., Khabele, D., Mahadevan-Jansen, A.	Using Raman spectroscopy to detect cervical dysplasia in minority populations	2010	*Index test does not fit inclusion criteria*	Electrical impedance spectroscopy (EIS)	
215	Vargis, E., Kanter, E., Kanter, G., Rao, G., Mahadevan-Jansen, A.	Proximity to disease has a significant impact on automated disease classification of normal tissues	2010	*Index test does not fit inclusion criteria*	Electrical impedance spectroscopy (EIS)	
216	Vargis, E., Webb, C. N., Paria, B. C., Bennett, K. A., Reese, J., Al-Hendy, A., Mahadevan-Jansen, A.	Raman spectroscopy: An effective method of detecting biochemical changes of the pregnant cervix	2011	*Index test does not fit inclusion criteria*	Electrical impedance spectroscopy (EIS)	
217	Vizet J., Rehbinder J., Deby S., Roussel S., Nazac A., Soufan R., Genestie C., Haie-Meder C., Fernandez H., Moreau F., Pierangelo A.	In vivo imaging of uterine cervix with a Mueller polarimetric colposcope	2017	*Irrelevent study type or subject*		
218	Walmer, D. K., Merisier, D., Littman, E., Rodriguez, G., Venero, N., Henderson, M., Katz, D., Edwards, R.	Portable colposcopy in low-resource settings	2004	*Irrelevent study type or subject*		
219	Wang, W., Zhao, J., Short, M., Zeng, H.	Real-time in vivo cancer diagnosis using Raman spectroscopy	2015	*Index test does not fit inclusion criteria*	Electrical impedance spectroscopy (EIS)	
220	Wang, X., Zheng, B., Li, S., Zhang, R., Mulvihill, J. J., Chen, W. R., Liu, H.	Automated detection and analysis of fluorescent in situ hybridization spots depicted in digital microscopic images of Pap-smear specimens	2009	*Index test does not fit inclusion criteria*	Electrical impedance spectroscopy (EIS)	
221	Wikstr om Shemer, E. A., Volsky, M., Calantzis, T., Kruger, F., Shemer, I.	Pocket-sized colposcope offers a highly cost-effective curative intervention at minimum infrastructural requirements	2012	*Irrelevent study type or subject*		
222	Winkler, J. L., Lewis, K., Aguila, R. D., Gonzales, M., Delgado, J. M., Tsu, V. D., Sellors, J. W.	Is magnification necessary to confirm visual inspection of cervical abnormalities? A randomized trial in Peru	2008	*Index test does not fit inclusion criteria*	Aviscope	optical magnification 4X
223	Winkler, J. L., Tsu, V. D., Bishop, A., Scott, R., Sellors, J. W.	Confirmation of cervical neoplasia using a hand-held, lighted magnification device	2003	*Index test does not fit inclusion criteria*	Aviscope	optical magnification 4X
224	Wright, T. C., Jr., Kuhn, L.	Alternative approaches to cervical cancer screening for developing countries	2012	*Irrelevent study type or subject*		
225	Wu, T., Qu, J., Cheung, T. H., Lo, K., Yu, M. Y.	Preliminary study of detecting neoplastic growths in vivo with real time calibrated autofluorescence imaging	2003	*Index test does not fit inclusion criteria*	Electrical impedance spectroscopy (EIS)	
226	Wu, Y., Xi, P., Qu, J., Cheung, T. H., Yu, M. Y.	Depth-resolved fluorescence spectroscopy of normal and dysplastic cervical tissue	2005	*Index test does not fit inclusion criteria*	Electrical impedance spectroscopy (EIS)	
227	Wulan, N., Rasool, N., Belinson, S. E., Wang, C., Rong, X., Zhang, W., Zhu, Y., Yang, B., Tresser, N. J., Mohr, M., Wu, R., Belinson, J. L.	Study of the diagnostic efficacy of real-time optical coherence tomography as an adjunct to unaided visual inspection with acetic acid for the diagnosis of preinvasive and invasive neoplasia of the uterine cervix	2010	*Index test does not fit inclusion criteria*	Electrical impedance spectroscopy (EIS)	
228	Ye, Jing, Cheng, Xiao-Dong, Cheng, Bei, Cheng, Yi-Fan, Chen, Xiao-Jing, Lu, Wei-Guo	MiRNA detection in cervical exfoliated cells for missed high-grade lesions in women with LSIL/CIN1 diagnosis after colposcopy-guided biopsy.	2019	*Irrelevent study type or subject*		
229	Yeates, K. E., Sleeth, J., Hopman, W., Ginsburg, O., Heus, K., Andrews, L., Giattas, M. R., Yuma, S., Macheku, G., Msuya, A., Oneko, O.	Evaluation of a Smartphone-Based Training Strategy Among Health Care Workers Screening for Cervical Cancer in Northern Tanzania: The Kilimanjaro Method	2016	*Index test does not fit inclusion criteria*	VIA/VILI/Smartphone	iphone base digital cervicocgraphy - abstract and mag not stated
230	Yeates, K., Sleeth, J., Heus, K., Macheku, G., Msuya, A., Ondondo, J., Achieng Oneko, O.	The use of an mHealth strategy to detect and treat cervical cancer in Tanzania	2015	*Index test does not fit inclusion criteria*	VIA/VILI/Smartphone	iPhone 5S (Apple,Cupertino, CA)
231	Yu, B., Nagarajan, V. K., Ferris, D. G.	Mobile fiber-optic sensor for detection of oral and cervical cancer in the developing world	2015	*Index test does not fit inclusion criteria*	Electrical impedance spectroscopy (EIS)	
232	Yu, B., Shah, A., Nagarajan, V. K., Ferris, D. G.	Diffuse reflectance spectroscopy of epithelial tissue with a smart fiber-optic probe	2014	*Index test does not fit inclusion criteria*	Electrical impedance spectroscopy (EIS)	
233	Zeng, Xianxu, Zhang, Xiaoan, Li, Canyu, Wang, Xiaofang, Jerwick, Jason, Xu, Tao, Ning, Yuan, Wang, Yihong, Zhang, Linlin, Zhang, Zhan, Ma, Yutao, Zhou, Chao	Ultrahigh-resolution optical coherence microscopy accurately classifies precancerous and cancerous human cervix free of labeling.	2018	*Index test does not fit inclusion criteria*	Confocal microscopy	
234	Zlatkov, V.	Possibilities of the TruScreen for screening of precancer and cancer of the uterine cervix	2009	*Index test does not fit inclusion criteria*	Truscreen	
