# Supplementary figures and images for: Screening test accuracy of portable devices that can be used to perform colposcopy for detecting CIN2+ in low- and middle-income countries: a systematic review and meta-analysis
Source: BMC Womens Health. 2020 Nov 16;20:253. doi: 10.1186/s12905-020-01121-3 (PMC7670616; doi:10.1186/s12905-020-01121-3)

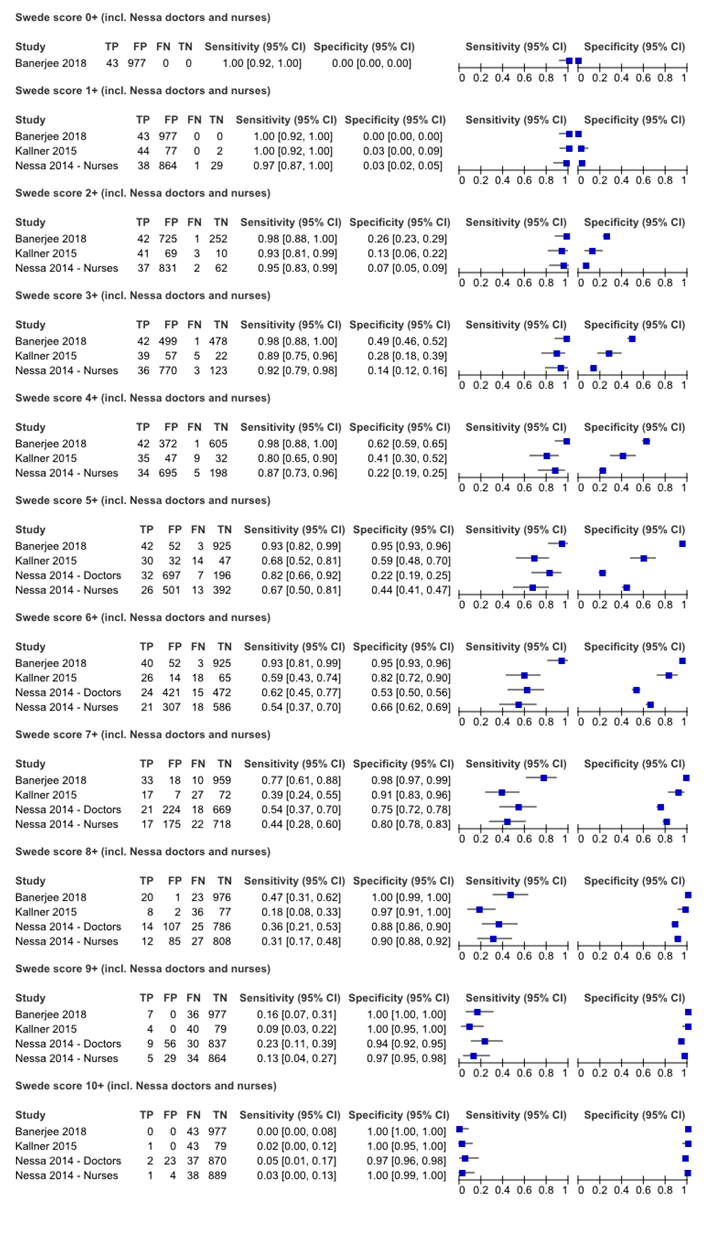

Supplement: Supplementary file 4 — Additional file 4. “Paired forest plot for all Swede score studies”. Sensitivity and specificity estimates for all Swede score thresholds. [file 12905_2020_1121_MOESM4_ESM.png]
