# Supplementary material for: Screening test accuracy of portable devices that can be used to perform colposcopy for detecting CIN2+ in low- and middle-income countries: a systematic review and meta-analysis
Source: BMC Womens Health. 2020 Nov 16;20:253. doi: 10.1186/s12905-020-01121-3 (PMC7670616; doi:10.1186/s12905-020-01121-3)
Supplement: Supplementary file 5 — Additional file 5. “Quality of the eligible studies”. Quality of eligible studies is scored using the QUADAS-2 criteria. [file 12905_2020_1121_MOESM5_ESM.docx]

**Supplementary material 6. Quality of the eligible studies as assessed by QUADAS-2 criteria**

| **Study** | **RISK OF BIAS** | | | | **APPLICABILITY CONCERNS** | | |
| --- | --- | --- | --- | --- | --- | --- | --- |
|  | **Patient selection** | **Index test** | **Reference standard** | **Flow and timing** | **Patient selection** | **Index test** | **Standard reference** |
| Banerjee 2018 | ? | L | L | L | L | L | L |
| Kallner 2015 | ? | H | L | L | L | L | L |
| Mueller 2018 | ? | L | H | L | L | L | L |
| Nessa 2014 | ? | H | H | L | L | L | L |
| Newman 2019 | ? | L | H | H | ? | L | L |

Risk of Bias and applicability concerns summary: author’s judgements about each domain for each included study, “H” high, “L” low, “?” unclear
